# The Quality of Grape Berries and Wine Is Enhanced Due to the Intercropping of Green Manure by Regulating Soil Microecology

**DOI:** 10.3390/foods15111923

**Published:** 2026-05-29

**Authors:** Qi Xie, Yue Wen, Pu Ren, Jianhong Cao, Jiakui Wang, Yulin Fang, Xiaofeng Yue, Yanlun Ju

**Affiliations:** 1 College of Enology, Northwest A&F University, Yangling 712100, China; xieqi2000@nwsuaf.edu.cn (Q.X.); 2022056250@nwsuaf.edu.cn (Y.W.); 13934758054@163.com (P.R.); fangyulin@nwsuaf.edu.cn (Y.F.); 2 Shangri-La Wine Company Limited, Diqing 674400, China; 3 College of Food Science and Engineering, Northwest A&F University, Yangling 712100, China

**Keywords:** grapes quality, wine quality, green manure, intercropping, soil, microecology

## Abstract

Planting green manure between rows is an excellent green orchard cultivation practice. However, there is a lack of research on the application of such measures in vineyards. In this study, the ‘Beibinghong’ grape was used as experimental material, and clear tillage was used as a control. The effects of intercropping rape and pea between rows for two consecutive years on soil microecology and grape and wine quality were studied. The main results were as follows: intercropping green manure increased the berry pH. Intercropping green manure differentially modulated phenolic profiles in grape berries: pea intercropping significantly increased total phenolic and tannin contents relative to clean tillage across both years, whereas rapeseed intercropping showed variable effects depending on phenolic class and vintage. Green manure treatments also altered the accumulation of aldehydes, alcohols, and terpenoids. The intercropping of green manure could effectively reduce soil temperature and maintain soil moisture in the surface soil layer, reduce soil pH and electrical conductivity, and increase soil microbial biomass, aggregate amount, enzyme activity and soil fertility. Intercropping green manure changed soil microbial diversity and community structure. At the phylum level, the relative abundances of *Chloroflexi* (bacteria) and *Mortierellomycota* (fungi) were significantly increased. At the genus level, the genera *Plectosphaerella* and *Alternaria*—both dominant saprotrophic fungi—were also significantly enriched. The results of a comprehensive evaluation of principal component and membership function and sensory evaluation showed that intercropping peas was the best strategy. Soil pH, electrical conductivity, nitrate content, LAP activity, and the phyla *Acidobacteriota*, *Chloroflexi*, and *Mortierellomycota* were significantly correlated with the acid content of wine, while soil enzyme activity was significantly correlated with the phenolic content of wine. These results indicated that intercropping green manure could drive the quality of grapefruits and wine by regulating soil nutrients, enzyme activities, basic physical and chemical properties, and microbial communities.

## 1. Introduction

The quality of grapes and the wine produced from them is strongly correlated, with grape cultivation practices exerting a profound influence on both [1]. For decades, clean tillage has been a favored traditional vineyard management approach, as it effectively controls weeds in the short term, reduces competition for water and nutrients between understory vegetation and grapevines, and enhances soil loosening and aeration [2]. However, despite these short-term benefits, long-term clean tillage results in bare inter-row soil within vineyards. This bare soil leads to diminished soil nutrient availability and biological activity, increased soil erosion, and disruption of soil microecology—consequences that conflict with the principles of sustainable modern agriculture [3].

In contrast, intercropping green manure between fruit tree rows or plants offers multiple ecological and agronomic advantages: it improves soil and water conservation, reduces reliance on chemical fertilizers and pesticides, regulates orchard microclimates, balances ecosystem functions, and ultimately contributes to enhanced fruit yield and quality [4]. Rapeseed and soybeans are among the most widely used green manure species for such intercropping systems [5]. Following green manure establishment, two key soil-related benefits emerge: first, the growth of green manure root systems increases soil porosity and decreases bulk density, facilitating the transport of soil nutrients and water [6]; second, the decomposition of green manure in soil promotes the formation of soil aggregates, which enhances soil water conductivity and water retention capacity [7]. Additionally, the ground cover provided by growing green manure helps maintain stable soil temperatures and reduces soil water evaporation—conditions that support healthy grapevine root development [8]. Green manure rotation also modifies soil chemical properties, including pH, organic matter content, and concentrations of nitrogen, phosphorus, and potassium [9]. These chemical changes, in turn, influence the soil’s internal energy exchange capacity and material cycling efficiency [10]—factors that are closely linked to grapevine growth and development [11,12].

Beyond soil physicochemical properties, intercropping (as a diversified planting strategy) enhances environmental biodiversity, promotes the proliferation of beneficial microbial communities, and fosters the development of antagonistic microbial taxa that suppress soil-borne diseases [13]. Soybeans (*Glycine max*), a representative legume, have been extensively studied for their nitrogen-fixing capacity and their ability to enhance rhizobial diversity and soil nitrogen status [14]. The rhizosphere of rapeseed plants, meanwhile, accumulates phosphorus-solubilizing microorganisms [15]. Rhizosphere microorganisms can indirectly alter soil acidity by decomposing organic matter, thereby influencing the ability of grapevine roots to absorb and translocate acidic compounds. Furthermore, the aggregation of beneficial microorganisms around grapevine roots accelerates the decomposition and turnover of soil nutrients, optimizing conditions for grapevine growth [16]. Peas (*Pisum sativum*), another leguminous cover crop, share the nitrogen-fixing trait with soybeans but differ in biomass turnover and root exudate chemistry. In this study, we selected rapeseed and pea to compare how leguminous versus non-leguminous green manures differentially affect vineyard soil microecology and grape-wine quality.

While green manure intercropping in vineyards has emerged as a promising direction for eco-friendly and efficient grape cultivation, critical knowledge gaps persist. Specifically, the impacts of this practice on soil nutrient dynamics, microbial community structure, and subsequent grape and wine quality remain incompletely understood. To address this gap, the present study systematically evaluated the effects of two consecutive years of intercropping green manure (rapeseed and peas) in vineyard rows on soil microecology and grape-wine quality. The findings demonstrate that green manure intercropping drives changes in soil microbial diversity and community composition, highlighting its significant potential for improving berry and wine quality.

## 2. Materials and Methods

### 2.1. Design of Experiment

The experiment was conducted at the Heyang Grape Experimental Demonstration Station (35°13′25′′ N, 110°11′59′′ E), Weinan City, Shaanxi Province, China. The soil is a sandy-gravelly mixture, characteristic of the region’s fluvial–aeolian depositional environment. It experiences a warm-temperate continental monsoon climate, with an annual mean temperature of 13.8 °C, a frost-free period of 196 days, annual sunshine duration of 2523.8 h, and mean annual precipitation of 488.85 mm—over 70% of which falls during the summer months (July–September). The test cultivar was ‘Beibinghong’, a wine grape variety bred from the cross between ‘Zuoyouhong’ and ‘84-26-53’. Grapevines were planted in 2012, with rows oriented north–south. The planting density was set at 1.0 m (plant spacing) × 2.5 m (row spacing). A fence-type trellis system was adopted, and the vines were trained to a single-trunk, two-arm tree shape.

The experiment was conducted from 2022 to 2024. Green manure was intercropped in vineyard inter-rows, with black mulch applied within rows. Two green manure species were used in the experiment: rape (*Brassica napus* L.), cultivar ‘Jinyou No.1’; and pea (*Pisum sativum* L.), cultivar ‘Texuan 604’. A randomized complete block design was adopted, including three treatments with three biological replicates each (plot size: ~30 m^2^): (1) Rape intercropping (YC): 1.5 m wide strips, seeding rate of 22.5 kg hm^−2^; (2) Pea intercropping (WD): 1.5 m wide strips, seeding rate of 150 kg hm^−2^; (3) Clean tillage (CK, control).

In early August 2022, rotary tillage was performed, followed by rape sowing; in late September 2022, rotary tillage and pea sowing were conducted. In mid-April 2023, rape was mowed, crushed, and incorporated into soil at full bloom, while peas were mowed and incorporated at the podding stage. The biomass and nutrient content of green manure are provided in Table S1. The above procedures were repeated from 2023 to 2024. During the experiment, conventional annual grape management practices were implemented, and no fertilizers were applied to the intercropped green manure.

The experiment followed a completely randomized design with four replications. Given the inherent spatial heterogeneity of open-field conditions, we took the following measures to control confounding: (i) the experimental site had a uniform cropping history (maize monoculture for the preceding three years with consistent fertilization regimes); (ii) all plots were located within a single contiguous field to avoid macro-scale soil variation; (iii) plot size was sufficiently large relative to the expected spatial autocorrelation range; and (iv) the coefficient of variation (CV) for the control treatment was less than 20%, suggesting acceptable within-field uniformity for this type of agronomic trial.

### 2.2. Collection of Samples

Five sampling points were taken in each plot of soil, and the samples obtained from these five points were mixed together to serve as the representative sample for that plot. Soil samples were collected from the 0–20 cm depth at each point, thoroughly mixed, and 1 kg of composite soil per biological replicate was obtained via the quartering method, then stored at 4 °C. After berry harvest, soil samples were collected from the 0–20 cm, 20–40 cm, and 40–60 cm depths in each plot and stored at 4 °C. For berries, except for the grapevines at the edge of the plot, uniformly sized and disease-free fruit clusters of the same quality were randomly selected at intervals of 3 m as the target fruit clusters; 400 to 500 berries were collected from the upper, middle, and lower parts of the tree canopy and shady and sunny areas of the target fruit clusters. The berries were carefully cut off with fruit branch shears and attached to the stems and stored at −40 °C. The fruit clusters from each processing method are harvested according to the industrial process and used as raw materials for winemaking, and wines are produced following the standard dry red wine protocol. All analyses were based on n = 3 biological replicates per treatment.

### 2.3. Analysis of Berry and Wine Quality

Titratable acidity, pH, reducing sugar, and soluble solids content were determined using an FTIR analyzer (Lyza 5000 Wine, Anton Paar Co., Ltd., Shanghai, China). Grape color was analyzed following Bai et al. [17] Ten berries were randomly selected per replicate to measure CIELab parameters (brightness L*, red/green coordinate a*, yellow/blue coordinate b*, chroma C*, and hue angle h°) with a wine color analyzer (W100, Hanon Advanced Technology Group Co., Ltd, Jinan, China), with three measurements taken per berry and averaged.

Glucose and fructose contents were determined following Li et al. [18] with slight modifications. Organic acid fractions were analyzed using the method of Ju et al. [19] with minor adjustments. Phenolic compounds were extracted from grape skins using a methanol-hydrochloric acid solution, and extracts were stored at −40 °C for subsequent analysis. Total phenolic content in grapes was determined via the Folin–Ciocalteu method and expressed as gallic acid equivalents. Tannin content in skins was analyzed using the methylcellulose precipitation method, expressed as catechin equivalents. Total flavan-3-ol content in skins was quantified via the p-DMACA-hydrochloric acid method. Total flavonoid content in grapes was determined following Pinelo et al. [20] with minor modifications and expressed as rutin equivalents.

The sensory evaluation test was conducted in the standard sensory analysis room. The evaluation team was composed of ten individuals with previous experience in wine tasting. They underwent three days of concentrated training on systematic aroma detection and tasting. The sensory quality of the wine was comprehensively evaluated using a 100-point scoring system, covering five aspects: color, clarity, aroma, taste, and typicality. The specific scoring table can be found in Text S1.

### 2.4. Volatile Compound Analysis

Volatile aroma compounds in grapes and wine were determined via headspace solid-phase microextraction coupled with gas chromatography-mass spectrometry (HS-SPME-GC-MS), following a published methodology [21] with modifications. Grape aroma extraction was performed according to Sanchezpalomo et al. [22] with slight adjustments.

GC-MS conditions were as follows: high-purity helium (99.9999%), 1 mL·min^−1^; 250 °C; HP-Innowax (60 m × 0.25 mm × 0.25 μm); held at 50 °C for 1 min, ramped to 220 °C at 3 °C·min^−1^, then held at 220 °C for 5 min; MS conditions: full-scan mode (mass range = 30–450 amu), electron ionization (EI) mode (70 eV), transfer line temperature = 280 °C, interface temperature = 240 °C, and ion source temperature = 230 °C.

Compounds were identified by matching spectra to the Shimadzu TQ8050NX mass spectral library. Quantification was performed via MS, and the content of each aroma compound (μg·L^−1^) was calculated using the internal standard method.

### 2.5. Determination of Soil Indices

During the active growth period of green manure, soil temperature and relative humidity at 0–5, 5–10, 10–15, and 15–20 cm depths were measured using a temperature-humidity recorder (RC-4HC, Jiangsu Jingchuang Electric Co., Ltd., Xuzhou, China) equipped with a metal probe. The amount of green manure incorporated into soil was calculated based on aboveground plant biomass [23]. Total nitrogen (TN) and total phosphorus (TP) contents were determined via an AA3 Continuous Flow Analytical System [10]; total potassium (TK) content was measured by flame photometry [24]; and total carbon (TC) content was assayed following Qiu et al. [25]. Soil physical properties were determined as follows: soil water content via the aluminum box drying method; field water-holding capacity via the ring knife soaking method; soil bulk density via the ring knife method; and soil aggregates via wet sieving.

Activities of soil enzymes—β-1,4-glucosidase (BG), β-D-cellobiohydrolase (CBH), β-1,4-N-acetylglucosaminidase (NAG), leucine arylamidase (LAP), and alkaline phosphatase (AKP)—were measured using microplate fluorescence analysis [26]. Soil acquisition enzyme activities were calculated following H. Ma et al.’s [27] method.

C-acq = (BG + CBH)/2

N-acq = (NAG + LAP)/2

P-acq = AKP

### 2.6. Soil Microbial Detection

Soil microbial DNA was extracted following Wechter et al. [28]. Total genomic DNA was isolated using the OMEGA Soil DNA Kit (M5635-02, Omega Bio-Tek, Norcross, GA, USA) per the manufacturer’s protocol and stored at –20 °C until analysis. PCR amplification targeted the bacterial 16S rRNA gene V3–V4 region (primers: 338F, 5′-ACTCCTACGGGAGGCAGCA-3′; 806R, 5′-GGACTACHVGGGTWTCTAAT-3′) and fungal ITS1 region (primers: ITS1F, 5′-CTTGGTCATTTAGAGGAAGTAA-3′; ITS2, 5′-GCTGCGTTCTTCATCGATGC-3′), with sample-specific 7 bp barcodes integrated into primers for multiplex sequencing.

PCR reactions contained 5 μL 5× buffer, 0.25 μL Fast pfu DNA Polymerase (5 U/μL), 2 μL dNTPs (2.5 mM), 1 μL each of forward/reverse primers (10 μM), 1 μL DNA template, and 14.75 μL ddH_2_O. Thermal cycling conditions: initial denaturation at 98 °C for 5 min; 25 cycles of 98 °C (30 s, denaturation), 53 °C (30 s, annealing), 72 °C (45 s, extension); final extension at 72 °C for 5 min.

PCR amplicons were purified with Vazyme VAHTSTM DNA Clean Beads (Vazyme, Nanjing, China) and quantified via the Quant-iT PicoGreen dsDNA Assay Kit (Invitrogen, Carlsbad, CA, USA). Amplicons were then pooled in equal amounts for paired-end 2 × 250 bp sequencing on the Illumina NovaSeq platform (NovaSeq 6000 SP Reagent Kit, Illumina, Inc., San Diego, CA, USA, 500 cycles) at Shanghai Personal Biotechnology Co., Ltd. (Shanghai, China).

Amplicon sequencing data were analyzed at multiple taxonomic levels. For clarity in reporting, priority was given to the genus level for differentially abundant taxa identified by LEfSe and correlation analysis. Where ASVs could not be reliably annotated to the genus level, the lowest confidently assignable taxonomic rank (e.g., family, order, or phylum) is explicitly stated in the text. Taxonomic ranks are indicated as follows: genus (*Alternaria*), family (*Pleosporaceae*), or phylum (*Chloroflexi*) upon first mention.

### 2.7. Statistical Analysis

#### 2.7.1. Grape and Wine Quality Parameters

Grape berry physicochemical traits—including yield, fruit size, soluble solids content (SSC), titratable acidity (TA), pH, organic acids, sugars, phenolic compounds, anthocyanins, and CIELab color parameters—and wine chemical parameters (alcohol, volatile acid, titratable acid, phenolics, anthocyanins, organic acids, and color) were analyzed to evaluate treatment effects and identify significant differences among clean tillage (CK), rape intercropping (YC), and pea intercropping (WD). Prior to parametric testing, all data were examined for normality using the Shapiro–Wilk test and for homogeneity of variance using Levene’s test. Because the assumptions of normality and homoscedasticity were satisfied, treatment effects were evaluated by one-way analysis of variance (ANOVA) using IBM SPSS Statistics (version 26), and Duncan’s multiple range test was applied as a post hoc comparison wherever ANOVA revealed a significant effect (*p* < 0.05) to identify specific differences among treatment means. Microsoft Excel 2019 was used solely for data organization; all inferential statistical computations were performed in SPSS.

#### 2.7.2. Volatile Aroma Compounds

Volatile aroma compound profiles (μg·L^−1^) in grape berries and wines, determined by HS-SPME-GC-MS, were subjected to partial least squares–discriminant analysis (PLS-DA) to identify treatment-discriminative aroma markers and visualize inter-treatment differences in volatile profiles. PLS-DA was performed to maximize covariance between the aroma compound matrix (X) and the treatment class matrix (Y), and key differential aroma compounds were selected based on variable importance in projection (VIP) scores > 1.0. Corresponding score and loading plots were generated using SIMCA 14.1.

#### 2.7.3. Soil Microecological Indicators

Soil physicochemical properties (temperature, moisture, pH, EC, water-stable aggregates, bulk density, field capacity, and nutrients), enzyme activities (BG, CBH, NAG, LAP, and AKP), and microbial community composition (bacterial 16S rRNA and fungal ITS amplicon sequencing data) were compared among treatments using the same parametric framework as in Section 2.7.1 (Shapiro–Wilk test, Levene’s test, one-way ANOVA, and Duncan’s test), while microbial community diversity patterns were visualized by principal coordinate analysis (PCoA) based on Bray–Curtis dissimilarity.

#### 2.7.4. Comprehensive Quality Evaluation and Soil-Quality Association

To integrate multi-trait information and rank overall grape and wine quality among treatments, principal component analysis (PCA) and membership function analysis were performed on the standardized 12 berry and 11 wine quality traits (Section 2.7.1) following Li [29]; principal components with eigenvalues > 1 were retained, and the factor loadings and principal component scores were used to calculate membership function values and comprehensive evaluation scores (D-values) for each treatment, with detailed formulas provided in Supplementary Text S1. To explore potential associations between soil microecological factors and fruit/wine quality, Pearson correlation analysis (two-tailed) was conducted between soil indicators (physicochemical properties, enzyme activities, and microbial community relative abundances at the phylum and genus levels; Section 2.7.3) and grape/wine quality parameters (Section 2.7.1); given the exploratory nature of this analysis and the limited sample size relative to the number of soil variables, regression-based modeling was not performed to avoid overfitting and multicollinearity artifacts. Correlation coefficients and significance levels (*p* < 0.05, *p* < 0.01) were visualized as heatmaps using Origin 2021.

## 3. Results

### 3.1. Grape Berry Quality

**Figure 1:**
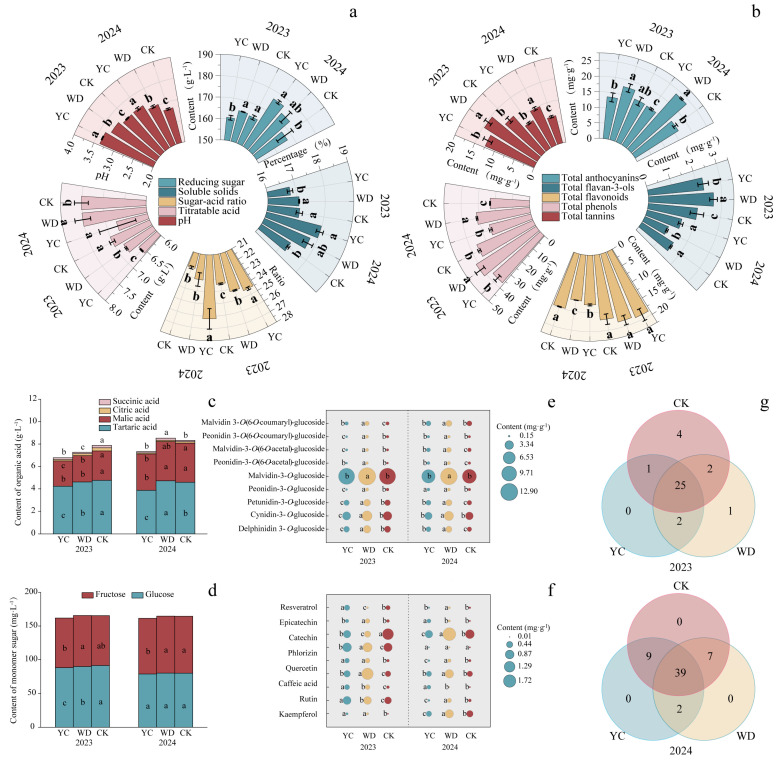
The physicochemical indicators of grape berries. (**a**) Basic indicators of grape berries. (**b**) The content of phenolic substances in grape berries. (**c**) Organic acid content in grape berries. (**d**) Sugar content in grape berries. (**e**) The content of monomeric anthocyanins in grape berries. (**f**) Monomeric phenol content in grape berries. (**g**) Venn diagram of volatile aroma substances in grape berries. Lowercase letters indicated significant differences (*p* < 0.05) between different treatments in the same year. Comparisons were not made across years.

As shown in Table 1, different treatments significantly affected fruit physical traits. One-way ANOVA F values for yield, hundred-grain weight, vertical diameter, transverse diameter, and fruit shape index were 0.408, 0.749, 2.437, 1.575, and 0.664 in 2023, and 1.845, 11.456 **, 2.432, 6.197, and 4.643 in 2024, respectively. * *p* < 0.05, ** *p* < 0.01, *** *p* < 0.001; ns, not significant. In 2023, the vertical diameter of berries in the rape (YC) treatment was significantly higher than that in clean tillage (CK) and pea (WD) treatments. Significant differences in fruit pH were observed among all treatments. For soluble solids content (SSC), YC showed no significant variation (<4.41% change) in 2024. Titratable acidity (TA) was highest in CK in 2023; in 2024, TA increased across all treatments, with YC significantly lower than other groups. Concentrations of succinic, citric, malic, and tartaric acids in YC were significantly lower than those in CK (Figure 1c). Regarding sugars: In 2023, YC had significantly lower reducing sugar content than other treatments; in 2024, YC’s reducing sugar was 5.52% higher than CK, but its fructose content was significantly lower than WD.

For phenolics and antioxidants (Figure 1b): In 2023, WD had the highest total phenols, total tannins, and total flavanols, while CK had the lowest (with no significant 15.88% improvement noted). In 2024, total phenols and tannins followed the order WD > YC > CK (WD significantly higher than CK); total flavonoids in YC and WD were lower than CK, while total flavanols in YC and CK were higher than WD. Nine monomeric anthocyanins were detected in ‘Beibinghong’ grape skins (Figure 1e,f), with 3-O-glucosides of delphinidin and cyanidin as the main components. Venn diagrams (Figure 1g) illustrate the overlap of volatile compounds detected across treatments. Notably, YC and WD shared 25 compounds in 2023 and 39 compounds in 2024 with CK, but each green manure treatment also harbored unique aroma markers in 2023: YC specifically contained Cedrol. These treatment-specific compounds, albeit minor in number, contributed to the distinct aroma profiles revealed by PLS-DA (Figures S1 and S2). WD had significantly higher contents of all anthocyanin monomers than other treatments. Over two years, YC showed higher catechin content, and WD had higher resveratrol, epicatechin, and quercetin contents than CK. Color difference values between YC/WD and CK ranged from 0.5 to 1.5, indicating weak color variation (Table S2).

HS-SPME-GC-MS analysis of fruit volatile aromas (Table S3) showed that in 2023, aromas were dominated by benzenoids, aldehydes, and alcohols; in 2024, alcohols, aldehydes, and ketones were the main classes. Hexanol and hexanal were the key compounds contributing to inter-treatment aroma differences.

### 3.2. Wine Quality

**Figure 2:**
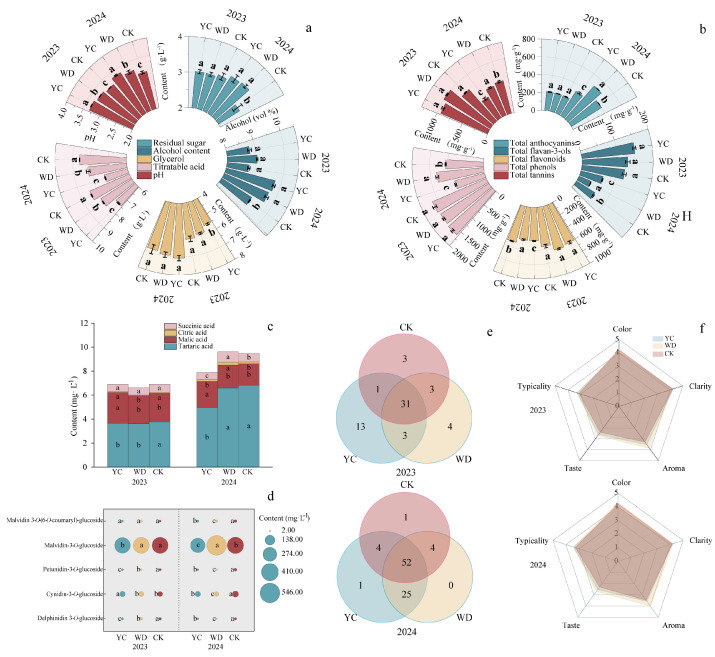
The physicochemical indicators of wine. (**a**) Basic chemical indicators of wine. (**b**) Content of phenolic substances. (**c**) Organic acid content. (**d**) Monomer anthocyanin content. (**e**) Venn diagram of volatile aroma substances in wine. (**f**) The sensory quality analysis radar chart of wine, each part is converted into a five-point rating based on its proportion of the total. Lowercase letters indicated significant differences (*p*  <  0.05) between different treatments in the same year. Year-to-year comparisons were not performed.

In both years, wine from green manure treatments showed significantly higher alcohol content than clean tillage (CK), which had the lowest alcohol content (≤11.53% and ≤14.85%, respectively). Volatile acid content across all treatments was <1.2 g·L^−1^, and titratable acid content was <4 g·L^−1^ (Figure 2a). CIELab parameters (Table 2) indicated that in 2023, all wines exhibited a purple hue, with rape (YC) and pea (WD) treatments darker than CK. In 2024, YC and WD wines were red, consistent with CK’s red color. In 2024, WD significantly increased total wine phenols and total flavonoids, while total tannin content was significantly reduced (Figure 2b). Over two years, malic acid content in YC wine was significantly lower than that in CK (Figure 2c).

Five monomeric anthocyanins were detected in wines. WD significantly reduced the contents of delphinidin-3-O-glucoside, cyanidin-3-O-glucoside, and petunidin-3-O-glucoside. Volatile aroma compounds of wines are detailed in Table S4. Key inter-treatment differences were observed in phenylethanol, isopentanol, isoamyl acetate, ethyl decanoate, ethyl hexanoate, ethyl laurate, and ethyl acetate. Concentrations of isoamyl alcohol, ethyl esters, and phenylethanol also varied among treatments.

Sensory evaluation results (Figure 2f) showed inter-treatment differences primarily in aroma and taste. Wines from green manure treatments had significantly higher aroma scores than CK, with more prominent aromas, better balance, and finer tannins. The WD treatment produced wine with the highest overall quality.

### 3.3. Soil Physicochemical Indicators

During green manure intercropping, daily average soil temperature at all depths was significantly lower in rape (YC) and pea (WD) treatments than in clean tillage (CK), with the most obvious differences (1–3 °C) at 0–5 cm and 5–10 cm; WD showed a significant cooling effect (Tables S5 and S7). No significant differences in daily average soil relative humidity were observed among treatments, despite consistent variation trends across depths (Tables S6 and S8).

**Figure 3:**
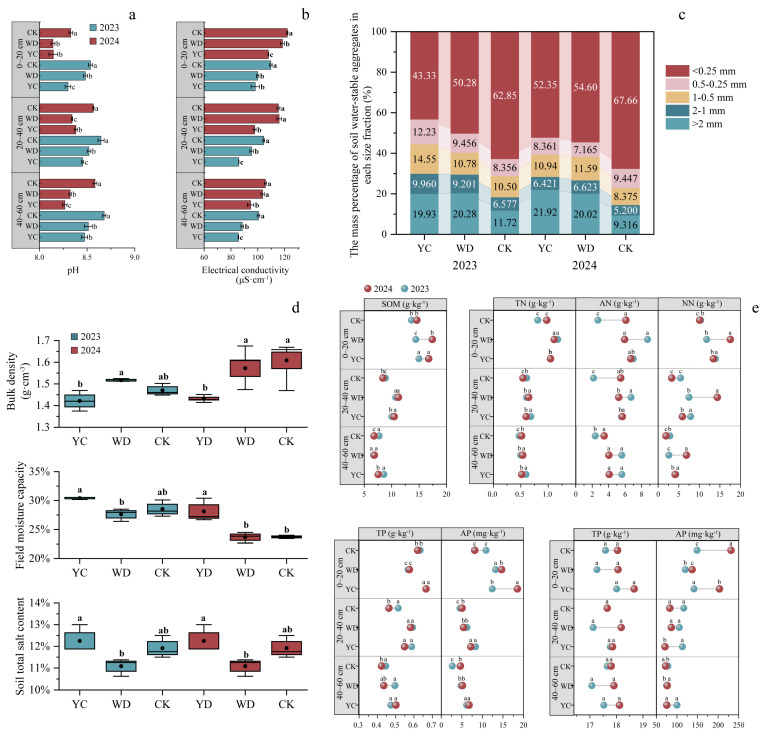
The influence of different intercropping patterns in vineyards on soil physicochemical indicators. (**a**) Soil pH at different soil depths. (**b**) Electrical conductivity at different soil depths. (**c**) Composition of soil water-stable aggregates within the 0–20 cm soil layer. (**d**) Soil physical properties. (**e**) Soil nutrients. Lowercase letters indicated significant differences (*p*  <  0.05) between different treatments in the same year.

Soil pH in all layers was significantly lower under YC and WD than CK. In 2023, 0–20 cm soil pH did not differ among treatments, but electrical conductivity (EC) was lower in YC and WD than in CK. In 2024, both pH and EC in 0–20 cm soil were lower in YC and WD than in CK, with pH reduced by 2.16–3.61% (Figure 1a,b). Differences in soil water-stable aggregates among treatments were driven by particles < 0.25 mm and 2 mm; large aggregates (>0.25 mm) were more abundant in YC and WD than CK (Figure 3c). In 2024, YC had higher soil water content and lower bulk density than CK (no 2023 differences between YC and WD), indicating long-term intercropping enhances soil water retention, nutrient holding, and permeability (Figure 3d). Soil organic matter (SOM), total nitrogen (TN), and total phosphorus (TP) were significantly higher in YC and WD than in CK; ammonium (NH_4_^+^-N) and nitrate (NO_3_^−^-N) also showed higher concentrations in YC and WD (Figure 3e).

### 3.4. Soil Microorganisms

**Figure 4:**
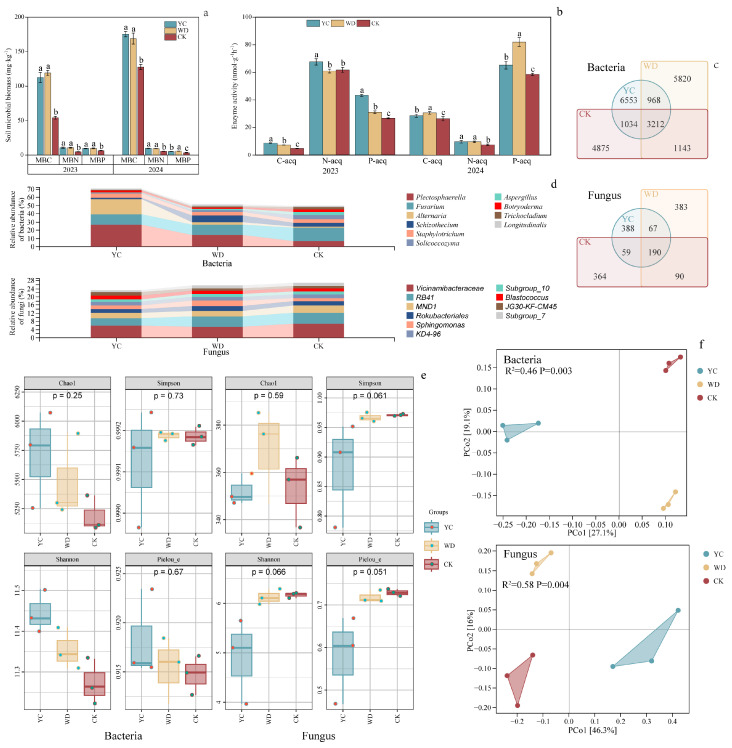
The influence of intercropping patterns among different vineyards on soil microorganisms. (**a**) The soil microbial biomass in the 0–20 cm soil layer. (**b**) Enzyme activities for C, N and P acquisition in the 0–20 cm soil layer. (**c**) Venn diagram of bacterial and fungal ASVs. (**d**) Relative abundance of the top bacterial and fungal taxa. Bacterial taxa are shown at the family or genus level as annotated; fungal taxa are shown at the genus level unless otherwise indicated (e.g., *Tremellomycetes*, class). Taxonomic rank is denoted in parentheses upon first mention in the text. (**e**) Alpha diversity of microbial communities. (**f**) Beta diversity of microbial communities. Lowercase letters indicated significant differences (*p*  <  0.05) between different treatments in the same year.

In 2023, microbial biomass C (MBC), N (MBN), and P (MBP) in the rape (YC) treatment increased by 107.86%, 124.58%, and 50.39%, respectively, compared to clean tillage (CK); in 2024, these increments were 37.36%, 77.84%, and 47.54%. Notably, YC’s MBC content was 55.85% higher in 2024 than in 2023, while MBN and MBP changes were insignificant (Figure 4a). For C, N, and P acquisition enzyme activities (Figure 4b), YC showed significantly higher activities than other treatments in 2023, whereas the pea (WD) treatment had the highest activities in 2024. Amplicon sequencing revealed 23,605 bacterial ASVs (Amplicon Sequence Variants) and 1541 fungal ASVs across all treatments. YC had the most bacterial ASVs (11,767, 14.64% more than CK), while WD had the most fungal ASVs (730, 3.84% more than CK). Unique ASVs followed the order YC > WD > CK for both bacteria and fungi (Figure 4c). At the genus level (Figure 4d), bacterial communities were dominated by the family *Vicinamibacteraceae*, the genus *RB41*, and the genus *MND1*, with the top ten genera accounting for < 30% of total relative abundance (indicating high species richness and even distribution). For fungi, YC had significantly higher relative abundances of *Plectosphaerella* (27.02%) and *Alternaria* (18.55%) (dominant genera) than other treatments, while CK had a significantly higher abundance of the genus Fusarium (16.16%) than YC and WD. Alpha diversity analysis (Figure 4e) showed that YC had a higher bacterial Chao1 index (species richness) than CK, and WD had a higher fungal Chao1 index. No significant inter-treatment differences were observed in the bacterial Simpson index, but YC’s bacterial Shannon index was significantly lower than CK’s. For fungi, YC’s Pielou index (species evenness) was significantly lower than WD and CK.

LEfSe analysis (LDA threshold = 3; Figure S5) identified 32 bacterial and 38 fungal taxonomic units with significant differences. YC had higher abundances of bacteria from the family *Micrococcaceae* and the class *Thermoleophilia*, while WD had higher abundances of *Sphingomonas* and the family *Comamonadaceae*. Fungal differences were more pronounced (LDA > 4): YC had *Pleosporaceae*, WD had the family *Lasiosphaeriaceae*, the genus *Schizothecium*, and the family *Cordycipitaceae*, and CK had the genus *Solicoccozyma*, the family *Piskurozymaceae*, the order *Filobasidiales*, and the class *Tremellomycetes*. PCoA showed the first two principal components explained 46.2% of bacterial and 62.3% of fungal community variation (Figure 4f). High intra-group similarity and clear inter-group separation confirmed distinct microbial community compositions among treatments.

### 3.5. Comprehensive Evaluation and Correlation Analysis

To evaluate grape and wine quality under different cultivation practices, 12 berry quality indexes and 11 wine quality indexes (including titratable acid and total phenols) were analyzed using principal component analysis (PCA) and membership function analysis over two years. For berry quality, PCA extracted two principal components with eigenvalues > 1, which explained 66.8% and 22.5% in 2023 and 53.1% and 40.3% in 2024 of the total variance, respectively, with a cumulative contribution of 89.3% and 93.4% (Table S7). For wine quality, the first two principal components cumulatively explained 96.2% and 97.3% of the variance (Table S8). These two components were deemed sufficient to represent the main effects of green manure intercropping on fruit and wine quality. Membership function values (D-values; Table 3) showed the pea (WD) treatment ranked first in berry quality in both years (scores: 0.87 and 0.66) and also topped wine quality rankings (scores: 0.79 and 0.68). These results confirm that pea green manure significantly improved wine quality.

**Figure 5:**
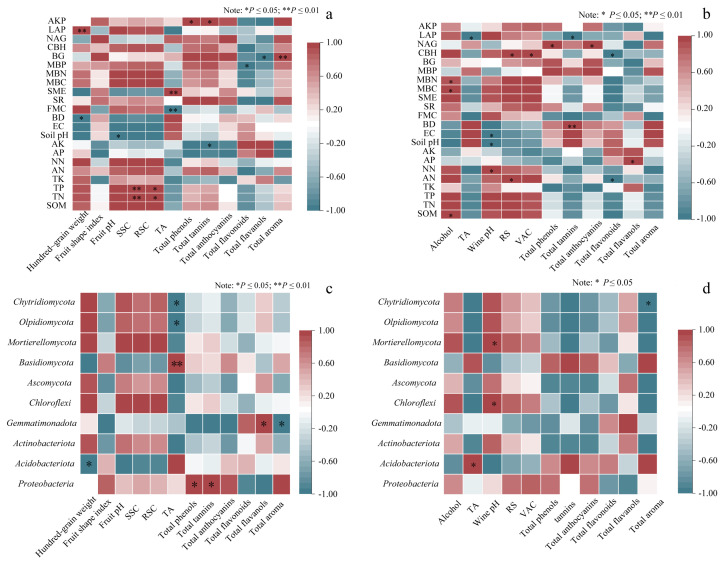
The grape berry and wine quality and soil ecological factor correlation analysis heat map. (**a**) Correlation analysis of grape berry quality and soil characteristics. (**b**) Correlation analysis of wine quality and soil characteristics. (**c**) Correlation analysis of grape berry quality and soil microorganisms. (**d**) Correlation analysis of wine quality and soil microbial characteristics. Bacterial taxa are reported at the phylum level (e.g., *Chloroflexi*, *Proteobacteria*), and fungal taxa at the genus level (e.g., *Alternaria*, *Mortierella*) unless reliable genus-level annotation was unavailable. * *p* < 0.05, ** *p* < 0.01.

Correlation analysis between soil properties (organic matter, enzyme activity, physicochemical properties, microorganisms) and quality indexes (12 fruit, 11 wine) was conducted, with results shown in Figure 5. The key correlations are described below.

Soil–fruit links: Soil nutrients, enzyme activity, and microorganisms strongly affected fruit sugar-acid content and phenolics. Reduced soil bulk density and OTA-producing fungus abundance increased fruit weight. Total nitrogen and phosphorus promoted grape sugar accumulation. Soil alkaline phosphatase activity and the phylum *Proteobacteria* abundance were significantly positively correlated with fruit total phenols and tannins. Field capacity, soil microbial biomass, the phylum *Basidiomycota*, and the phylum *Chytridiomycota* jointly influenced fruit titratable acid; β-1,4-glucosidase and the phylum *Gemmatimonadota* co-regulated fruit total flavanols and aroma compounds.

Soil-wine links: Soil organic matter, microbial biomass C, and microbial biomass N increased wine alcohol content—likely due to higher fruit sugar. Soil pH, electrical conductivity, nitrate-N, *Chloroflexi*, and *Mortierellomycota* jointly affected wine pH. Increased leucine aminopeptidase activity reduced wine titratable acid and total tannins; higher β-1,4-N-acetylglucosaminidase activity promoted wine total phenols and anthocyanins. Elevated β-D-cellobiohydrolase activity increased wine residual sugar and volatile acids while decreasing total flavonoids.

## 4. Discussion

### 4.1. Effect of Intercropping Green Manure on Grape Berry Quality

Different intercropping methods of green manure in the field had no significant effect on berry yield, which is consistent with the findings of previous studies [12]. Over the two-year experimental period, green manure intercropping exerted a minimal influence on grapefruit shape, size, and soluble solid content—another result that aligns with the research by Beslic [30]. Notably, intercropping with rapeseed green manure significantly reduced the titratable acid content of grape berries, resulting in a “high sugar and low acid” profile. In contrast, no significant difference in titratable acid content was observed between pea green manure intercropping and clear tillage; both treatments produced berries with “high sugar and high acid,” a characteristic conducive to wine aging. It is important to clarify that the ‘low acid’ profile of YC berries does not contradict the observed reduction in soil pH under green manure intercropping. Soil pH reflects the acidity of the rhizosphere environment, whereas berry titratable acidity is a physiological trait governed by organic acid synthesis, degradation, and dilution during fruit ripening. In the present study, YC treatment exhibited stronger soil cooling effects (Table S5), which may have delayed fruit ripening and enhanced respiratory acid degradation, resulting in lower malic and tartaric acid concentrations despite a more acidic soil environment. Conversely, WD treatment maintained higher berry acidity, likely due to its leguminous nitrogen-fixing capacity altering the timing and balance of sugar-acid accumulation. These findings underscore that soil–fruit quality linkages are mediated by complex physiological and environmental interactions and that correlation patterns (Figure 5) should be interpreted as hypothesis-generating rather than causally definitive. Intercropping with either rape or pea green manure increased the total phenol and tannin contents in grape berries while also inducing differences in berry color. Among the treatments, pea green manure intercropping led to the highest anthocyanin content in berries, though no significant variations in total flavonoid and total flavanol contents were detected across all treatments. With the increase in green manure cover years, the accumulation of aromatic substances in grape berries was gradually affected, though this effect varied significantly between years [31]. The shift from benzenoid-dominant aromas in 2023 to ketone-dominant profiles in 2024 may reflect year-specific climatic conditions (e.g., temperature, precipitation timing) or the cumulative effects of two consecutive years of green manure incorporation on soil nitrogen cycling and fruit ripening dynamics. Specifically, green manure intercropping significantly impacted the accumulation of aldehydes, terpenoids, and alcohols—key components of grape aromatic profiles.

A comprehensive evaluation of grape berry quality revealed that green manure intercropping generally improved the fruit quality of ‘Beibinghong’ grapes, with pea green manure intercropping yielding the most favorable results. Correlation analysis between grape berry quality and soil ecological factors further indicated that soil total nitrogen and total phosphorus contents were significantly positively correlated with berry soluble solid and reducing sugar contents, confirming that improved soil fertility can enhance fruit sugar accumulation. This analysis also showed that soil alkaline phosphatase activity and the relative abundance of *Proteobacteria* were significantly positively correlated with berry total phenol and tannin contents, suggesting that soil nutrient cycling promotes the accumulation of phenolic substances in grapes. Additionally, soil field water-holding capacity, metabolic entropy (qCO_2_), and the relative abundances of *Basidiomycota*, *Olpidiomycota*, and *Chytridiomycota* all affected berry titratable acid content, indicating that soil microbial activity can influence acid accumulation in grape berries.

### 4.2. Influence of Planting Green Manure on WINE Quality

“Terroir” refers to the topography, soil properties, and climatic conditions of a specific region. The terroir characteristics of vineyards are closely associated with the sensory quality of wine [32], and green manure intercropping can indirectly influence wine quality by modifying soil properties. Bouzas-Cid et al. [33] reported that after two years of intercropping ryegrass, white clover, and natural grass in the rows of ‘Mencia’ grapevines, natural grass and ryegrass intercropping significantly increased total anthocyanin content in wine compared with clear tillage—a finding that supports the link between cover crops and wine quality.

In the present study, wines from rapeseed and pea green manure intercropping treatments exhibited higher alcohol content and pH, as well as lower titratable acid content, than those from clear tillage. This pattern corresponded to the sugar and acid profiles of the grape berries used for winemaking, confirming a direct connection between berry composition and wine chemical properties. With respect to phenolic compounds, wines from pea green manure intercropping had higher total phenol and anthocyanin contents than clear tillage wines. In contrast, wines from rapeseed green manure intercropping showed lower total phenol, tannin, and anthocyanin contents than clear tillage wines—differences that contributed to distinct color variations among treatments. Specifically, rapeseed green manure intercropping resulted in rose–red wine, pea green manure intercropping produced purple–red wine, and clear tillage yielded wine–red wine. Combined with the determination of monomeric anthocyanin contents, these results suggest that green manure intercropping may alter wine color by influencing the content of delphinidin-3-O-glucoside in wine [34].

Across all treatments, tartaric acid and malic acid were the dominant organic acids, with relatively high concentrations. Sensory evaluation results further indicated that the wines exhibited an acidic taste, a characteristic that implies high aging potential. With regard to monomeric phenols, phenolic acids were the primary components in wines from all treatments: caffeic acid accounted for a large proportion of monomeric phenols in 2023, while protocatechuic acid was the major component in 2024. Aroma is a key indicator of wine quality and style, shaped by grape variety, terroir, and winemaking processes [35]. Xi et al. found that native grass cover increased the aroma substance content of Cabernet Sauvignon dry red wine, imparting more floral and sweet ripe fruit aromas [36]. Similarly, Peng et al. [37] demonstrated that green manure cover in vineyard rows enhances wine aroma. In the current study, alcohols, esters, and benzenes were the main aroma substances in ‘Beibinghong’ wines across all treatments. Green manure intercropping significantly affected the accumulation of alcohols and esters, introducing black fruit and floral aromas to the wines. Additionally, wines from green manure intercropping treatments had a softer taste, more balanced sugar-acid ratios, and more delicate tannins—results that were consistent with the findings of principal component analysis and membership function analysis.

### 4.3. Effects of Intercropping Green Manure on Soil Microecology

Green manure exerts a significant influence on soil temperature, moisture, physical–chemical properties, nutrient content, microbial community, and orchard microclimate, thereby reshaping the soil microecosystem. Prior studies have demonstrated that green manure regulates air and soil temperature and humidity, enhances air and soil stability, and facilitates the growth and development of fruit tree roots as well as the utilization of soil water and fertilizers [37]. Over the two-year study period, the 0–20 cm soil temperature in plots intercropped with rape and pea green manure was lower than that in clear tillage plots, which aligns with findings from previous research [38]. No significant difference in soil moisture was observed between the two groups. This can be attributed to the dual effects of green manure: while grass cover contributes to soil moisture retention, green manure competes with fruit trees for soil moisture during spring and summer droughts [39]. Green manure roots penetrate the soil, increasing soil porosity. After incorporation into the field, plant residues promote the formation of soil aggregates and improve the capacity for water and nutrient transport. Specifically, intercropping with rape green manure significantly reduced surface soil bulk density and increased field water-holding capacity and soil aggregate content, consistent with the results reported by Wang et al. [40]. In contrast, intercropping with pea green manure exerted a moderate effect, which may be associated with differences in green manure species. Soil pH and electrical conductivity are key indicators of soil salinization. Song et al. found that intercropping significantly reduced soil salinity and electrical conductivity, a result similar to that of the present study [41]. Numerous studies have shown that green manure cultivation increases soil organic matter and total nitrogen content [42]. Additionally, Ma et al. reported that leguminous green manure has a more pronounced effect on soil nitrate content than non-leguminous green manure, a finding that was further confirmed in this study [43]. A limitation of the present study is the absence of blocking or baseline soil characterization. While we attempted to minimize field heterogeneity through site selection and uniform management, unmeasured soil variables may have contributed to residual variance. Future trials should incorporate a randomized complete block design and pre-experiment soil sampling to further partition treatment effects from edaphic background variation.

The introduction of new interspecific relationships and organic carbon sources via orchard green manure cultivation significantly alters the original soil microecology. Compared with clear tillage or bare soil, green manure mulching or grass cultivation has been shown to significantly increase soil microbial biomass carbon and nitrogen contents while modifying soil microbial activity [44]. In the present study, intercropping with rape and pea green manure significantly increased soil microbial biomass C, N, and P, soil respiration intensity, and the activities of soil C, N, and P-acquiring enzymes. These results indicate that green manure incorporation enhances soil microbial metabolic activity and promotes soil C, N, and P cycling. Soil enzyme activities differed significantly between 2023 and 2024, with P-acquiring enzymes maintaining high activity throughout the two-year period. According to soil enzyme stoichiometry studies, a 1:1:1 ratio of C, N, and P-acquiring enzyme activities is most favorable for soil nutrient absorption and utilization; deviations from this ratio may lead to nutrient limitation [45]. This observation may be related to the long-term C and N limitation in the test soil. Soil metabolic entropy (qCO_2_), defined as the ratio of soil respiration intensity to microbial biomass carbon, represents the respiration intensity per unit of soil microorganisms. A higher qCO_2_ value indicates that a larger proportion of carbon sources is used for microbial respiration rather than being stored as microbial biomass carbon to improve soil nutrient levels [46]. Compared with clear tillage, intercropping with green manure did not increase soil metabolic entropy, suggesting that the soil had a strong carbon sequestration capacity and could effectively enhance soil carbon storage.

Kim et al. found green manure mulching significantly increased the richness and diversity of soil bacterial and fungal communities compared with clear tillage [47]. In the present study, intercropping with pea green manure did not significantly increase bacterial community diversity but tended to increase bacterial richness. This may be due to the insufficient duration of green manure cultivation, consistent with the results of Qu et al. [48]. In contrast, intercropping with rape green manure significantly reduced the diversity and richness of the soil fungal community. This phenomenon may be explained by the fact that the rape used in this experiment was of the mustard type, which produces high levels of glucosinolates and isothiocyanates after incorporation into the field—compounds with strong inhibitory effects on soil fungi [49]. Principal component analysis, cluster analysis, and correlation analysis of surface soil properties and soil microorganisms revealed that *Chloroflexi* and *Mortierellomycota* possess organic matter decomposition and denitrification functions. The increased relative abundance of these taxa may contribute to the elevation of soil nitrate content. Different green manure species exerted distinct effects on soil properties and microorganisms. Rape green manure significantly reduced soil bulk density, electrical conductivity, and pH, leading to the concentration of dominant fungal community groups. In contrast, pea green manure significantly increased soil enzyme activities and total nutrient contents. These results suggest that the selection of appropriate green manure species should be based on specific agricultural production conditions.

It is important to acknowledge the limitations of inferring causality from the soil-quality correlations presented in this study. First, the observational nature of the two-year field trial precludes definitive causal conclusions: while we observed significant associations between soil pH, electrical conductivity, and Chloroflexi abundance with wine acidity (Figure 5), these relationships may reflect co-responses to green manure intercropping rather than direct mechanistic links. Second, soil physicochemical and microbial variables are inherently intercorrelated (e.g., pH covaries with nutrient availability and microbial community composition), which introduces multicollinearity that would compromise the reliability of regression coefficients had such models been attempted. Third, the relatively small sample size (three replicates per treatment) limits statistical power for complex multivariate modeling. We therefore employed Pearson correlation as an exploratory, hypothesis-generating tool to identify candidate soil factors warranting further investigation, rather than as a confirmatory test of causal pathways. Future research should combine structural equation modeling (SEM) or path analysis with expanded replication and temporal sampling to disentangle direct and indirect effects of soil microecological factors on grape and wine quality.

## 5. Conclusions

This study investigated the effects of two green manure intercropping strategies (rapeseed and pea) on vineyard soil, grape berries, and wine over two consecutive years, with clear tillage as the control. The key findings are as follows:

Green manure intercropping did not significantly affect grape yield and had minimal impacts on fruit shape, size, and basic coloration but significantly influenced the accumulation of sugars, acids, phenolics compounds (notably total phenols and tannins under pea intercropping), and aromatic substances in grape berries. Notably, the dominant volatile aroma classes differed between vintages: benzenoids, aldehydes, and alcohols prevailed in 2023, whereas alcohols, aldehydes, and ketones dominated in 2024, with hexanol and hexanal serving as key inter-treatment discriminators in both years. These changes further altered the contents of organic acids, phenolic acid monomers, alcohols, and esters in the resulting wine. Correlation-driven observations revealed that increased soil alkaline phosphatase activity and *Proteobacteria* abundance contributed to higher total phenol and tannin contents in grape berries, while soil pH, electrical conductivity, nitrate content, and the relative abundances of *Chloroflexi* and *Mortierella* affected wine pH.

Green manure intercropping improved vineyard soil microecology and fertility while enhancing grape berry and wine quality—with pea green manure exhibiting the most pronounced positive effects. Specifically, green manure reduced soil temperature and maintained soil moisture during the grape-growing period. After incorporation into the soil, green manure alleviated soil salinization, increased microbial metabolic activity in the surface soil, and promoted soil carbon (C), nitrogen (N), and phosphorus (P) cycling. Among the two green manure types, rapeseed intercropping increased soil bacterial community diversity but decreased fungal community diversity, leading to the concentration of dominant fungal taxa.

Comprehensive evaluation of grape berry and wine quality indicated that pea green manure intercropping is the optimal row-intercropping strategy for the studied vineyard system. Collectively, these results demonstrate that green manure intercropping modulates grape berry and wine quality by regulating soil nutrient status, enzyme activities, basic physicochemical properties, and microbial community structure.

## Figures and Tables

**Table 1:** Effects of different treatments on grape yield and berry physical properties.

Years	Treatments	Yield per Tree (kg)	Hundred-Grain Weight (g)	Vertical Diameter (mm)	Transverse Diameter (mm)	Fruit Shape Index
2023	YC	2.99 ± 0.12 a	119.72 ± 1.32 a	12.31 ± 0.87 b	12.21 ± 0.78 b	1.01 ± 0.03 a
	WD	3.13 ± 0.11 a	119.12 ± 1.21 a	13.00 ± 0.94 a	12.89 ± 0.93 a	1.01 ± 0.03 a
	CK	3.05 ± 0.18 a	118.74 ± 1.45 a	12.41 ± 0.77 b	12.26 ± 0.82 b	1.01 ± 0.02 a
2024	YC	3.28 ± 0.06 a	129.13 ± 1.20 a	13.72 ± 0.51 a	13.75 ± 0.70 a	1.00 ± 0.02 b
	WD	3.29 ± 0.10 a	122.59 ± 0.88 b	13.74 ± 0.35 a	13.16 ± 0.32 b	1.04 ± 0.02 a
	CK	3.19 ± 0.03 a	125.65 ± 2.49 b	13.22 ± 0.24 b	13.10 ± 0.46 b	1.01 ± 0.04 a

Lowercase letters indicated significant differences (*p*  <  0.05) between different treatments in the same year.

**Table 2:** Effects of different treatments on the color of wine.

Years	Treatments	L*	a*	b*	c*	h°	ΔE	Color Fitting
2023	YC	30.02 ± 0.40 b	53.79 ± 0.13 b	21.39 ± 0.28 b	57.88 ± 0.23 c	0.38 ± 0.01 a	7.17 ± 0.34 a	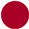
	WD	32.61 ± 0.33 a	58.67 ± 0.09 b	21.08 ± 0.14 b	62.34 ± 0.10 b	0.35 ± 0.01 c	2.44 ± 0.16 b	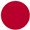
	CK	32.91 ± 0.26 a	60.14 ± 0.09 a	22.99 ± 0.12 a	64.38 ± 0.12 a	0.37 ± 0.01 b	0.00 ± 0.00 c	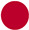
2024	YC	42.99 ± 0.16 a	55.57 ± 0.19 c	10.71 ± 0.13 c	55.63 ± 0.18 b	0.05 ± 0.00 c	10.17 ± 0.09 b	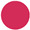
	WD	31.81 ± 0.58 c	61.07 ± 0.47 b	23.12 ± 0.20 a	65.30 ± 0.41 a	0.36 ± 0.01 a	13.09 ± 0.51 a	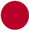
	CK	38.64 ± 0.09 b	64.57 ± 0.16 a	12.52 ± 0.12 b	65.77 ± 0.13 a	0.19 ± 0.00 b	0.00 ± 0.00 c	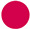

Lowercase letters indicated significant differences (*p*  <  0.05) between different treatments in the same year.

**Table 3:** Comprehensive evaluation of grape and wine quality under different treatments. y1 and y2: principal component scores for PC1 and PC2. u1 and u2: membership function values (0–1 scale) derived from y1 and y2, respectively. D value: comprehensive evaluation score calculated as the variance-weighted sum of u1 and u2 (D = u1 × W1 + u2 × W2), where weights reflect the relative contribution of each principal component. Rank: quality ranking based on descending D value (1 = highest overall quality). Detailed formulas are provided in Supplementary Text S2.

Sample	Years	Treatment	y1	y2	u1	u2	D Value	Rank
grape	2023	YC	2.06	−1.91	1.00	0.00	0.58	2
		WD	0.91	2.70	0.77	1.00	0.87	1
		CK	−2.97	−0.62	0.00	0.28	0.12	3
	2024	YC	−0.56	2.56	0.34	1.00	0.62	2
		WD	2.87	−0.87	1.00	0.19	0.66	1
		CK	−2.31	−1.69	0.00	0.00	0.00	3
wine	2023	YC	2.34	−1.42	1.00	0.00	0.71	2
		WD	0.75	2.00	0.71	1.00	0.79	1
		CK	−3.10	−0.59	0.00	0.24	0.07	3
	2024	YC	−2.60	−1.21	0.00	0.00	0.00	3
		WD	−0.06	2.34	0.48	1.00	0.68	1
		CK	2.65	−1.13	1.00	0.02	0.63	2

## Data Availability

The original contributions presented in this study are included in the article/Supplementary Materials. Further inquiries can be directed to the corresponding authors.

## Supplementary Materials

The following supporting information can be downloaded at https://www.mdpi.com/article/10.3390/foods15111923/s1, Text S1: Wine sensory evaluation form; Text S2: The trait indicators of each group on each principal component were standardized using the membership function; Table S1: The amount and nutrient content of green manure under different treatments; Table S2: Effects of relay cropping green manure on the color of grape berry; Table S3: Effects of intercropping green manure on volatile aroma substances in grape berry; Table S4: Effects of intercropping green manure on volatile aroma substances in wine; Table S5: Effects of relay cropping green manure on daily average soil temperature; Table S6: Effects of relay cropping green manure on soil daily average relative humidity; Table S7: Principal component analysis of grape berry quality under different treatments; Table S8: Principal component analysis of wine quality under different treatments; Figure S1: PLS-DA analysis of volatile aroma substances of berris in 2023. a: Replacement test, b: biplot, c: VIP; Figure S2: PLS-DA analysis of volatile aroma substances of berris in 2024. a: Replacement test, b: biplot, c: VIP; Figure S3: PLS-DA analysis of volatile aroma substances of wine in 2023. a: Replacement test, b: biplot, c: VIP; Figure S4: PLS-DA analysis of volatile aroma substances of wien in 2024. a: Replacement test, b: biplot, c: VIP; Figure S5: LEfSe analysis of soil microbial communities under different treatments.
