# Supplementary material for: The Quality of Grape Berries and Wine Is Enhanced Due to the Intercropping of Green Manure by Regulating Soil Microecology
Source: Foods. 2026 May 29;15(11):1923. doi: 10.3390/foods15111923 (PMC13256945; doi:10.3390/foods15111923)
Supplement: Supplementary file 1 [file foods-15-01923-s001.zip › foods-4321141-supplementary.pdf]

**Supplementary materials**

Text S1. Wine sensory evaluation form

1) The higher the score, the greater the intensity or quality.

2) The color is rated from 1 to 5. The color tones of red wine are as follows: (1) purple-red, (2) ruby red, (3) pomegranate red, (4) reddish-brown, (5) dark red.

| Evaluation index | | Score |
|---|---|---|
| Visual（10） | Colour（5） | |
| | Clarity（5） | |
| Aroma（30） | Characteristics（6） | |
| | Intensity（8） | |
| | Condition（16） | |
| Taste（40） | Purity（10） | |
| | Intensity（10） | |
| | Prolongation（10） | |
| | Quality（10） | |
| Global evaluation（20） | Typicality（20） | |
| Conclusions（100） | | |

Text S2

The trait indicators of each group on each principal component were standardized using the membership function, $y1 = \Sigma PC_1 Z_j$, $y2 = \Sigma PC_2 Z_j$. Through $u(X_j)=(X_j-X_{min})/(X_{max}-X_{min})$, the membership functions of y1 and y2 were obtained as u1 and u2, and then the comprehensive evaluation value D was calculated using the weight $W_j = P_j / \sum_{j=1}^{n} P_j$, $D = \sum_{j=1}^{n} [u(X_j) * W_j]$.

Table S1 The amount and nutrient content of green manure under different treatments

| Years | Green manure | Biomass ($kg \cdot hm^{-2}$) | TN ($g \cdot kg^{-1}$) | TP ($g \cdot kg^{-1}$) | TK ($g \cdot kg^{-1}$) | TOC ($g \cdot kg^{-1}$) | C/N |
|---|---|---|---|---|---|---|---|
| 2023 | Rape | 7959.26±154.09 a | 23.44±0.40 b | 1.97±0.07 b | 14.58±0.22 b | 446.33±0.81 a | 19.04±0.33 a |
| | Pea | 5972.96±308.35 b | 28.78±0.28 a | 3.22±0.40 a | 19.73±0.12a | 444.70±1.87 a | 15.45±0.16 b |
| 2024 | Rape | 7695.83±317.79 a | 31.19±0.76 b | 3.75±0.05 a | 34.21±0.53 a | 421.24±9.63 b | 13.50±0.45 a |
| | Pea | 7508.33±269.35 a | 34.54±0.47 a | 3.38±0.05 b | 30.46±0.38 b | 441.93±4.13 a | 12.79±0.21 b |

Note: Lowercase letters indicated significant differences ($P < 0.05$) between different treatments in the same year.

.

Table S2 Effects of relay cropping green manure on the color of grape berry.

| Years | Treatments | *L** | *a** | *b** | *c** | *h°* | *ΔE* |
|---|---|---|---|---|---|---|---|
| 2023 | YC | 27.41±1.07 a | 1.27±0.08 a | 0.82±0.16 ab | 1.52±0.11 a | 0.57±0.09 b | 1.32±0.42 a |
| | WD | 28.44±1.23 a | 0.84±0.09 b | 0.90±0.16 a | 1.23±0.16 b | 0.81±0.08 a | 0.68±0.22 b |
| | CK | 27.66±0.94 a | 0.74±0.07 c | 0.69±0.07 b | 1.01±0.06 c | 0.75±0.08 a | 0.00±0.00 c |
| 2024 | YC | 27.29±0.73 b | 1.09±0.14 b | 0.90±0.16 b | 1.42±0.13 b | 0.69±0.12 b | 1.38±0.31 a |
| | WD | 27.61±0.59 ab | 1.38±0.20 a | 0.92±0.15 b | 1.66±0.23 a | 0.59±0.06 c | 1.10±0.40 a |
| | CK | 28.30±1.07 a | 0.98±0.12 b | 1.34±0.20 a | 1.66±0.19 a | 0.94±0.08 a | 0.00±0.00 c |

Note: Lowercase letters indicated significant differences ($P < 0.05$) between different treatments in the same year.

Table S3 Effects of intercropping green manure on volatile aroma substances in grape berry.

| | Category | Volatile aroma substances | YC | WD | CK |
|---|---|---|---|---|---|
| 2023 | Alcohols | Hexan-1-ol | 13.62±0.79b | 86.19±1.82a | 9.91±0.65c |
| | | 2-Ethylhexan-1-ol | 300.94±17.9ab | 275.02±17.34b | 324.35±24.13a |
| | | Nonan-1-ol | - | - | 12.96±0.29 |
| | | Furan-2-ylmethanol | - | - | 13.15±2.25 |
| | | Octan-1-ol | 122.8±8.61a | 121.8±8.19a | 129.33±12.1a |
| | | Dodecan-1-ol | 60.57±4.68c | 121.71±0.76a | 86.02±4.56b |
| | Aldehydes | 2-Methylpropanal | 21.08±1.92a | 13.92±1.69a | 22.09±8.1a |
| | | 2-Methylbutanal | 45.85±2.51a | 10.5±1.44c | 27.3±1.21b |
| | | Hexanal | 39.69±4.31a | 14.95±1.26c | 32.18±2.46b |
| | | Nonanal | 85.1±1.29a | 25.17±2.73c | 75.14±2.8b |
| | | Furan-2-carbaldehyde | 313.19±18.93a | 311.3±12.41a | 303.95±11.45a |
| | | Decanal | 33.26±2.23a | 16.23±0.97b | 19.95±2.16b |
| | | 5-Methylfuran-2-carbaldehyde | 27.63±0.78a | 25.02±1.85a | 25.49±1.51a |
| | Esters | Ethyl acetate | - | - | 140.88±6.55 |
| | | Ethyl (E)-but-2-enoate | 34.99±0.57a | 33.34±3.08a | - |
| | | Heptyl formate | - | 10.7±0.87b | 26.87±1.09a |
| | Ketones | Butane-2,3-dione | 45.08±0.87a | 24.95±0.92b | 25.07±2.57b |
| | | 3-Hydroxybutan-2-one | 13.83±1.94a | 7.83±0.5b | - |

| | | | | | |
|---|---|---|---|---|---|
| | Terpenoids | 6,10-Dimethylundeca-5,9-dien-2-one | 24.69±0.44b | 31.57±3.36a | 26.39±2.04b |
| | | 1-(Furan-2-yl)ethan-1-one | 27.81±2.54b | 40.39±3.09a | 17.74±1.72c |
| | | 3,7-Dimethylocta-1,6-dien-3-ol | 25.06±0.71a | 23.64±2.82a | 25.58±1.43a |
| | | 3,7-Dimethyloct-6-en-1-ol | - | 25.87±1.88a | 17.44±0.88b |
| | | 2-(4-Methylcyclohex-3-en-1-yl)propan-2-ol | 22.58±1.67a | 10.36±1.19c | 16.99±1.1b |
| | | Cedrol | - | 19.86±1.5a | - |
| | | 2-Methyl-5-(propan-2-yl)phenol | - | - | 116.9±8.31 |
| | Benzenoids | Toluene | 28.26±2.63a | 20.47±1.03b | 23.78±3.41ab |
| | | Methyl benzoate | 10.71±1.45a | 9.91±0.28a | 11.7±2.65a |
| | | 2-Methylbenzaldehyde | 322.26±22.28a | 329±13.73a | 344.02±6.81a |
| | | 1,4-Dimethoxybenzene | 254.63±29.32a | 226.39±11.48a | 217.84±15.04a |
| | | Methyl 2-hydroxybenzoate | 264.12±13.87a | 241.56±2.22b | 249.78±7.78ab |
| | | 2,4-Dimethylbenzaldehyde | 52.74±1.88b | 73.5±6.42a | 65.63±2.74a |
| | | 1-(2,4-Dimethylphenyl)ethan-1-one | 139.49±6.07a | - | 148.84±11.82a |
| | | Phenylmethanol | 159.72±4.98a | 152.52±13.47a | 155.27±19.71a |
| | | 2-Phenylethan-1-ol | 112.94±26.33a | 74.34±3.65b | 75.97±2.71b |
| | | Benzaldehyde | 122.83±9.29ab | 120.64±2.64b | 136.77±7.61a |
| 2024 | Alcohols | 2-Methylbut-3-en-2-ol | 1.97±0.26a | 1.53±0.19ab | 1.24±0b |
| | | 2,5-Dimethylhexane-2,5-diol | - | 0.21±0.05a | 0.16±0.02a |
| | | Hexan-1-ol | 144.61±14.25a | 165.07±18.21a | 167.72±21.54a |

| | | | | |
|---|---|---|---|---|
| | (Z)-Hex-3-en-1-ol | 2.85±0.32b | 3.14±0.28b | 24.27±3.11a |
| | (E)-Hex-2-en-1-ol | - | 146.53±14.82a | 140.09±16.57a |
| | (Z)-Hex-2-en-1-ol | 1.75±0.08b | 2.04±0.11a | 1.29±0.02c |
| | Oct-1-en-3-ol | 15.06±1.32a | 8.45±0.65b | 7.5±1.35b |
| | 2-Ethylhexan-1-ol | 10.22±0.93ab | 10.81±1.3a | 7.27±0.99b |
| | Octan-1-ol | 4.78±0.44a | 4.64±0.52a | 3.59±0.4a |
| | Nonan-1-ol | 3.99±0.03a | - | 3.22±0.03b |
| | 3,6-Nonadien-1-ol | 0.41±0.01a | 0.3±0.03b | - |
| | 2,3-Dimethylpentanal | 0.27±0.02a | 0.3±0.02a | 0.26±0.01a |
| | 2-Methylpropanal | 0.64±0.02a | 0.74±0.11a | 0.53±0.04a |
| | Butanal | 1.87±0.38ab | 1.94±0.18a | 1.11±0.03b |
| | 2-Methylbutanal | 1±0.02b | - | 2.86±0.18a |
| | 3-Methylbutanal | 3.87±0.07a | 3.73±0.11ab | 3.31±0.23b |
| | Pentanal | 0.53±0b | 0.73±0.05a | 0.75±0.05a |
| | Hexanal | 870.2±56.08a | 942.4±94.46a | 422.05±38.83b |
| | (E)-Pent-2-enal | 0.32±0.06a | 0.43±0.08a | 0.26±0.04a |
| | Heptanal | 3.4±0.12a | - | 2.83±0.42a |
| | (E)-Hex-2-enal | 61.91±5.14a | 73.24±5.61a | 55.5±8.02a |
| | Hex-2-enal | 1844.2±159.24a | 2192.3±208.46a | 1885.25±250.77a |
| Aldehydes | Octanal | 3.88±0.72a | 3.89±0.79a | 2.88±0.23a |

| | | | | |
|---|---|---|---|---|
| | (E)-Hept-2-enal | 1.93±0.19b | 2.86±0.32b | 5.27±0.51a |
| | Nonanal | 17.03±2.46a | 17.25±3.04a | 13.11±0.78a |
| | (E,E)-Hexa-2,4-dienal | 8.7±0.75a | 9.59±1.13a | 6.85±0.85a |
| | (E,E)-Hepta-2,4-dienal | - | 6.74±0.66a | 5.52±0.51a |
| | Decanal | 25.05±1.46a | 21.47±3.99ab | 14.51±1.26b |
| | (E)-Non-2-enal | 3.97±0.65a | - | 0.41±0.06b |
| | (E,Z)-Nona-2,6-dienal | 1.41±0.21a | 1.11±0.11ab | 0.85±0.16b |
| | β-Cyclocitral | 1.33±0.02b | 3.9±0.2a | 1.21±0.28b |
| | Ethyl acetate | 12.78±0.3b | 22.94±0.46a | 20.18±1.62a |
| | Methyl butyrate | 0.48±0.09a | 0.42±0.01a | 0.46±0.02a |
| | Butyl acetate | 0.68±0.04a | 0.62±0.12a | 0.62±0.12a |
| Esters | Butyl acrylate | 0.14±0b | 0.21±0.01a | 0.15±0.02b |
| | Hexanoic acid | 7.82±0.69a | 7.02±0.28a | 6.73±0.3a |
| Acids | (E)-Hex-2-enoic acid | 4.01±0.35a | 4.32±0.56a | - |
| | Octan-3-one | 0.21±0.01ab | 0.19±0.01b | 0.27±0.04a |
| | 2,2,6-Trimethylcyclohexanone | 0.34±0a | 0.39±0.06a | 0.3±0.01a |
| | Octane-2,3-dione | 1.75±0.15a | 1.52±0.25a | 0.9±0.15b |
| | 6-Methylhept-5-en-2-one | 10.95±0.67a | 8.93±1.11a | 7.9±1.12a |
| | Isophorone | 2.6±0.33b | 9.72±0.92a | 1.09±0.15c |
| Ketones | β-Damascenone | 14.78±1.83b | 280.56±37.28a | 206.39±18.06a |

| | | | | |
|---|---|---|---|---|
| | 4-(2,6,6-Trimethylcyclohex-1-en-1-yl)but-3-en-2-one | 0.82±0.1a | - | 0.79±0.07a |
| Ethers | Lime oxide | - | 1.69±0.2a | 1.14±0.16b |
| | (E)-Linalool oxide | 7.46±1.23b | - | 26.6±2.47a |
| | Linalool | 13.35±1.4a | 15.35±1.87a | 7.68±0.79b |
| | Dihydrolinalool | 13.16±2.19ab | 14.01±0.15a | 8.9±1.15b |
| | α-Terpineol | 2.27±0.32b | 3.86±0.24a | 1.59±0.26b |
| Terpenoids | (E)-Nerolidol | 0.35±0.02b | 2.47±0.24a | 0.44±0.01b |
| | o-Xylene | 0.52±0.07b | 9.54±0.69a | 0.45±0.07b |
| | Acetophenone | 0.77±0.07a | - | 0.41±0.01b |
| | 1,1,6-Trimethyl-1,2-dihydronaphthalene | - | 6.7±1.12a | 4.36±0.43b |
| | Phenylmethanol | 10.57±1.15a | - | 5.44±0.28b |
| | 2-Phenylethan-1-ol | - | 10.1±1.59a | 4.77±0.27b |
| | α-Calacorene | - | 14.79±2.6a | 5.13±0.46b |
| Benzenoids | Phenylacetaldehyde | 2.58±0a | - | 2.53±0.19a |

Note: Lowercase letters indicated significant differences ($P < 0.05$) between different treatments in the same year. “ - ”indicates not checked.

Table S4 Effects of intercropping green manure on volatile aroma substances in wine.

| | Category | Volatile aroma substances | YC | WD | CK |
|---|---|---|---|---|---|
| 2023 | Esters | Ethyl 3-hydroxybutyrate | 83.49±3.7 | - | - |
| | | Ethyl (S)-lactate | 2533.91±228.84a | 466.42±20.56b | 271.98±23.44b |
| | | Ethyl hexyl benzoate | 40.46±0.29 | - | - |
| | | Diethyl succinate | 1079.12±14.32a | 1020.99±24.86a | 731.79±24.73b |
| | | Ethyl butyrate | 1419.09±65.09a | 561.9±9.52b | 334.1±24.44c |
| | | Methyl decanoate | 928.4±18.96a | 438.68±18.83b | 231.51±12.9c |
| | | Ethyl decanoate | 60964.77±1776.35a | 36611.62±1341.06b | 15961.41±481.45c |
| | | Isoamyl decanoate | 1123.38±66.46a | 568.08±13.01b | 270.44±15.47c |
| | | Ethyl nonanoate | 460.7±25.26 | - | - |
| | | Ethyl myristate | 1237.83±29.37 | - | - |
| | | Ethyl pentadecanoate | - | 1770.96±185.78 | - |
| | | Ethyl undecanoate | - | 438.92±27.13 | - |
| | | Methyl octanoate | 948.37±30.85a | 571.31±20.75b | 397.72±16.51c |
| | | Ethyl octanoate | 75020.75±3259.3a | 72277.89±1833.05a | 38001.16±453.87b |
| | | Isobutyl octanoate | 119.42±8.85b | 148.63±16.84a | - |
| | | Isoamyl octanoate | - | 1217.05±90.96a | 652.99±55.69b |

| | | | | |
|---|---|---|---|---|
| | Hexyl acetate | 547.64±30.51a | 554.97±39.25a | 385.64±14b |
| | Octyl acetate | 123.54±32.91 | - | - |
| | Ethyl acetate | 12246.28±770.55a | 8572.62±280.1b | 6241.12±550.37c |
| | Isopropenyl acetate | 111.45±7.02b | - | 29.59±0.2a |
| | Isobutyl acetate | 755.75±42.76a | 290.65±25.01b | 246.12±22.51b |
| | Isoamyl acetate | 33188.72±1822.9a | 10404.53±194.99b | 9370.15±650.78b |
| | Ethyl isovalerate | 373.7±19.32a | 172.66±5.77b | 125.63±6.33c |
| | Ethyl laurate | 14012.07±808.58a | 9338.69±340.09b | 3746.08±261.99c |
| | Ethyl hexanoate | 20723.84±1624.96a | 15320.35±608.3b | 10412.63±483.7c |
| | Ethyl palmitate | 2429.67±194.06 | - | - |
| | Butane-2,3-dione | 348.98±6.19a | 229.03±4.32b | 160.82±13.97c |
| Ketones | 3-Hydroxybutan-2-one | 478.28±15.23 | - | - |
| | Hydroxyacetone | 418.38±16.05 | - | - |
| Terpenoids | Nerol | - | 79.98±6.48 | - |
| | (E)-Nerolidol | - | 59.29±5.95 | - |
| | Butyric acid | 346.69±13.28a | 290.64±9.49b | 181.28±8.6c |
| | Hexanoic acid | 4639.18±271.68a | 3446.32±169.17b | 2514.82±95.64c |
| Acids | Octanoic acid | 5174.8±291.55a | 2785.08±182.17b | 1321.17±91.15c |
| | Isobutyric acid | 2808.84±60.36a | 2793.77±7.24a | 2161.3±6.93b |
| | Decanoic acid | 1678.94±46.69a | 218.87±8.94b | |

| | | | | |
|---|---|---|---|---|
| Aldehydes | Furan-2-carbaldehyde | 84.4±3.26 | - | - |
| Alcohols | Nonan-1-ol | 338.22±20.77a | 365.04±28.82a | 229.62±19.18b |
| | 3-Methylpentan-1-ol | - | 53.49±4.14a | 58.67±6.2a |
| | 3-Methylthiopropan-1-ol | 664.1±11.9b | 768.4±47.96a | 353.27±24.82c |
| | 2,6-Dimethylheptan-4-ol | - | - | 125.11±11.1 |
| | (E)-Hex-2-en-1-ol | 120.62±10.8 | - | - |
| | (E)-Hex-3-en-1-ol | 127.71±4.2b | 152.09±3.93a | 138.13±7.02b |
| | Dodecan-1-ol | 224.92±5.53a | 159.19±16.05b | - |
| | Undecan-1-ol | - | - | 86.37±4.19 |
| | Octan-1-ol | 985.41±13.15a | 678.22±12.79b | 447.5±25.97c |
| | (Z)-Hex-3-en-1-ol | 89.75±6.98a | - | - |
| | 2-Methylpropan-1-ol | 17405.6±225.42a | 17233.7±822.68a | 11588.79±747.37b |
| | 3-Methylbutan-1-ol | 156524.56±4083.14a | 158610.48±2622.65a | 102756.33±6430.79b |
| | Propan-1-ol | 632.18±9.2a | 503.1±17.39b | 408.08±22.1c |
| | Butan-1-ol | 45.66±2.00 | - | - |
| | Decan-1-ol | 607.59±45.8a | 294.46±23.69b | 204.34±18.98c |
| | Hexan-1-ol | 2697.79±214.49b | 3176.82±132.73a | 2927.71±85.25ab |
| Benzenoids | Phenol | 55.15±5.06a | - | - |
| | Benzaldehyde | - | 160.09±15.98a | 150.38±18.12a |
| | 2-Phenylethan-1-ol | 34943.93±2475.47b | 54794.34±2519.92a | 28335.33±515.27c |

| | | | | | |
|---|---|---|---|---|---|
| | | Methyl salicylate | - | - | 78.95±2.02 |
| | | Phenethyl acetate | 2158.73±151.81b | 2488.03±63.65a | 1240.67±107.55c |
| 2024 | Esters | Ethyl 2-methylbutyrate | 25.69±1.43a | 24.95±3.19a | - |
| | | Ethyl (Z)-hex-3-enoate | 9.15±0.84ab | 10.36±0.55a | 7.75±0.48b |
| | | Ethyl propionate | 1545.24±151.74a | 1575.55±66.73a | - |
| | | Isoamyl propionate | 63.52±1.33a | 52.56±3.57b | - |
| | | Diethyl succinate | 129.51±11.27a | - | 149.16±2.57a |
| | | Ethyl butyrate | 453.93±3.65a | 377.06±23.52b | - |
| | | Isoamyl butyrate | 10.87±0.75b | 11.53±1.27b | 15.13±1a |
| | | Ethyl heptanoate | 42.24±0.76a | 35.67±2.67ab | 29.38±3.03b |
| | | Methyl decanoate | 310±12.1b | 412.49±4.97a | 297.4±10.38b |
| | | Ethyl decanoate | 11079.54±695.07b | 14664.23±236.49a | 12067.56±1166.12b |
| | | Isobutyl decanoate | 14.99±0.78a | 15.69±1.29a | 8.51±0.58b |
| | | Isoamyl decanoate | 141.65±5.02a | 170.84±12.75a | 171.68±8.87a |
| | | Methyl hexanoate | 48.77±0.13b | 71.47±5.17b | 105.99±12.42a |
| | | Isoamyl hexanoate | 127.87±6.14a | 118.57±1.42a | - |
| | | Heptyl formate | 140.53±10.41a | 112.92±0.39b | - |
| | | Ethyl lactate | 45.56±0.28b | 47.78±0.49a | - |
| | | Ethyl myristate | 195.82±19.04a | 221.18±8.24a | 224.74±6.52a |
| | | Ethyl pentadecanoate | 21.26±1.33b | 31.62±2.24a | 25.92±0.03b |

| | | | | |
|---|---|---|---|---|
| | Propyl octanoate | 92.08±2.42a | 64.56±6.07b | - |
| | Hexyl octanoate | 18.05±1.22a | 9.95±0.57b | - |
| | Methyl octanoate | 477.23±7.01c | 566.03±21.75b | 669.33±7a |
| | Ethyl octanoate | 23511.82±863.74b | 26148.93±1099.98ab | 29137.3±1387.26a |
| | Isoamyl octanoate | 454.82±27.37b | 461.1±2.52b | 618.76±5.82a |
| | Ethyl 9-decenoate | 105.42±10.26a | 106.83±9.7a | 102.64±3.45a |
| | Propyl acetate | 113.86±4.21a | 112.81±10.67a | 52.51±5.34b |
| | Hexyl acetate | 472.96±24.64b | 964.85±77.22a | - |
| | Methyl acetate | 7.74±0.82b | 11.6±0.14a | - |
| | Octyl acetate | 25.99±0.75b | 98.56±5.93a | - |
| | Ethyl acetate | 1936.6±265.85b | 2899.78±109.47a | 1415.38±112.24b |
| | Isobutyl acetate | 98.37±5.4b | 132.96±9.08a | - |
| | Isoamyl acetate | 4940.04±69.93b | 7416.44±622.31a | 4088.03±105.88c |
| | Ethyl isovalerate | 79.19±2.91a | 65.09±3.64b | - |
| | Methyl laurate | 106.57±7.57b | 122.88±1.32b | 179.26±13.01a |
| | Ethyl laurate | 2504.43±145.09b | 3305.45±51.68a | 2777.94±99.57b |
| | Ethyl hexanoate | 7059.74±57.05b | 8301.89±633.16ab | 9026.17±471.53a |
| | Methyl palmitate | 17.96±3.88a | 20.76±0.56a | - |
| | Ethyl palmitate | 484.53±32.11a | 412.08±6.97b | 466.98±10.13ab |
| Ketones | Butane-2,3-dione | 113±0.12a | 120.29±5.22a | - |

| | | | | |
|---|---|---|---|---|
| | Pentane-2,3-dione | 47.34±2.87b | 70.96±6.66a | 30.93±3.78c |
| | Hexane-2,3-dione | - | 25.62±2.31a | 12.5±0.34b |
| | Heptan-2-one | 6.13±0.51 | - | - |
| | 3-Hydroxybutan-2-one | - | 11.54±0.15b | 20.98±0.32a |
| | β-Damascenone | 837.79±33.87b | 761.79±6.78b | 983.12±26.3a |
| | 6-Methylhept-5-en-2-one | 13.12±0.51a | 10.24±0.01b | 11.42±0.95ab |
| | 2-Undecanone | 24.16±2.52a | 21.95±0.99a | 19.08±1.06a |
| Terpenoids | (E)-β-Farnesene | 8.28±0.24b | 9.7±0.09ab | 11.16±1.31a |
| | α-Farnesene | 5±0.09b | 4.02±0.32b | 6.28±0.6a |
| | α-Terpineol | - | 37.65±2.47b | 59.45±0.47a |
| | Dihydrolinalool | 71.75±5.82a | - | 40.21±0.11b |
| | Farnesol | 11.37±1.25a | 14.06±0.69a | 12.9±2.69a |
| | (E)-Nerolidol | 25.64±2.9c | 47.07±0.82b | 120.59±8.29a |
| | Linalool | 122.77±10.31a | 130.46±9.53a | 128.73±2.1a |
| | Terpinolene | 54.45±3.51a | 48.42±3.38a | - |
| | Citronellol | 116.42±9.52a | 96.05±0.64a | 94.95±6.72a |
| | Citronellyl acetate | 22.58±1.23a | 22.89±1.89a | - |
| Acids | Heptanoic acid | 61.1±2.67b | 67.11±1.36a | - |
| | Hexanoic acid | 1036.95±89.15b | 952.3±13.04b | 1251.95±16.36a |
| | Octanoic acid | 3680.19±72.78c | 3878.51±18.74b | 5537.34±38.79a |

| | | | | |
|---|---|---|---|---|
| | Isobutyric acid | 150.74±8.39b | 97.31±4.76b | 271.11±52.23a |
| Aldehydes | Decanal | 308.73±26.66a | 201.22±15.77b | 209.62±13.64b |
| | 3-Methylbutanal | 4.33±0.7a | 4.57±0.07a | - |
| | Octanal | - | - | 431.14±36.42 |
| Ethers | 1-Ethoxypropan-2-ol | 87.18±6.99a | 80.52±3.8a | 76.53±6.49a |
| | Lime oxide | 8.31±0.61b | 8.62±0.39b | 15.29±0.22a |
| Alcohols | Tetradecan-1-ol | 28.89±1.48b | 26.16±0.43b | 45.52±0.6a |
| | Oct-1-en-3-ol | 51.81±4.97a | 45.81±2.12a | - |
| | Nonan-2-ol | 55.82±5.74a | 38.73±2.52b | - |
| | 3-Methylpentan-1-ol | 134.65±3.84b | 130±7.14b | 153.19±3.78a |
| | 3-Methylthiopropan-1-ol | 123.01±9.56a | 120.08±7.97a | 113.67±5.64a |
| | 4-Methylpentan-1-ol | 80.28±0.19b | 88.8±5.93b | 125.58±4.81a |
| | (E)-Hex-2-en-1-ol | 13.89±2.44a | 5.87±0.14b | - |
| | Octan-1-ol | 422.41±32.77b | 558.77±27.45a | 375.04±26.51b |
| | (Z)-Hex-3-en-1-ol | 116.57±10.29a | 94.66±3.42b | 60.67±1.1c |
| | 2-Methylpropan-1-ol | 2020.58±7.69a | 1929.54±129.35a | 1482.32±98.84b |
| | 3-Methylbutan-1-ol | 27298.51±1090.51a | 27118.49±1799.61a | 28653.74±2854.98a |
| | Propan-1-ol | 549.1±13.01a | 580.75±32.69a | - |
| | Decan-1-ol | 190.4±17.1a | 187.8±0.52a | 192.64±11.23a |
| | Hexan-1-ol | 1939.43±77.45a | 1393.72±74.88b | 1105.59±132.13b |

| | | | | |
|---|---|---|---|---|
| | 1,1,6-Trimethyl-1,2-dihydronaphthalene | 120.92±8.82c | 201.9±8.58b | 432.5±17.88a |
| | 4-Allylphenol | 4.83±0.28a | - | 2.21±0.01b |
| | α-Calacorene | 167.06±17.3a | 144.28±2.13a | 35.4±0.52b |
| | Phenylmethanol | 121.5±5.3a | 102.25±3.25b | 88.35±5.23b |
| Benzenoids | 2-Phenylethan-1-ol | 8458.64±400.26b | 8091.74±390.92b | 14313.27±656.71a |
| | Ethyl cinnamate | - | 5.96±0.34a | 5.5±0.13a |
| | Ethyl 3-hydroxy-3-phenylpropanoate | 11.2±0.52b | - | 24.67±1.64a |
| | Phenethyl acetate | 526.69±16.25c | 757.4±20.72b | 1047.92±52.78a |
| | Benzyl acetate | 17.07±0.53b | 21.54±0.57a | - |

Note: Lowercase letters indicated significant differences ($P < 0.05$) between different treatments in the same year. “ - ”indicates not checked.

Table S5 Effects of relay cropping green manure on daily average soil temperature.

| Year | Soil depth（cm） | Treatment | Temperture（℃） | | | | | | | | | | | |
|---|---|---|---|---|---|---|---|---|---|---|---|---|---|---|
| | | | Day1 | Day2 | Day3 | Day4 | Day5 | Day6 | Day7 | Day8 | Day9 | Day10 | Day11 | Day12 |
| 2023 | 0-5 | YC | 17.39 | 21.50 | 21.82 | 16.50 | 19.74 | 17.57 | 12.18 | 13.73 | 17.44 | 12.91 | 14.89 | 17.45 |
| | | WD | 16.37 | 20.44 | 21.33 | 15.97 | 16.38 | 15.60 | 11.94 | 13.68 | 16.71 | 12.70 | 14.48 | 17.03 |
| | | CK | 18.02 | 21.87 | 22.38 | 16.72 | 19.99 | 19.86 | 14.15 | 17.19 | 17.97 | 15.02 | 17.83 | 19.90 |
| | 5-10 | YC | 16.26 | 20.41 | 21.25 | 16.08 | 18.55 | 15.78 | 12.14 | 13.67 | 16.67 | 12.93 | 14.41 | 17.07 |
| | | WD | 14.61 | 17.71 | 19.33 | 15.66 | 16.76 | 15.93 | 13.13 | 13.21 | 15.31 | 13.20 | 14.24 | 16.22 |
| | | CK | 16.52 | 20.50 | 21.34 | 17.07 | 18.44 | 19.64 | 14.62 | 15.82 | 17.03 | 17.22 | 17.22 | 17.34 |
| | 10-15 | YC | 15.39 | 17.04 | 19.24 | 17.12 | 17.39 | 17.10 | 15.23 | 15.26 | 17.10 | 15.05 | 15.66 | 17.62 |
| | | WD | 13.78 | 15.36 | 17.28 | 15.91 | 15.97 | 15.84 | 14.49 | 14.12 | 15.24 | 14.36 | 14.46 | 15.50 |
| | | CK | 15.77 | 17.81 | 20.13 | 17.17 | 17.23 | 17.16 | 15.03 | 15.54 | 17.22 | 14.93 | 15.91 | 17.83 |
| | 15-20 | YC | 14.90 | 16.21 | 18.05 | 17.94 | 17.82 | 17.24 | 14.67 | 15.21 | 16.34 | 15.38 | 15.38 | 16.68 |
| | | WD | 13.58 | 14.89 | 16.71 | 15.93 | 15.79 | 15.86 | 15.02 | 14.50 | 15.40 | 14.73 | 14.63 | 15.49 |
| | | CK | 14.66 | 15.90 | 17.98 | 18.97 | 17.72 | 17.06 | 16.98 | 14.73 | 15.66 | 16.67 | 14.91 | 15.90 |

| Year | Soil depth（cm） | Treatment | Temperture（℃） | | | | | | | | | | | |
|---|---|---|---|---|---|---|---|---|---|---|---|---|---|---|
| | | | Day1 | Day2 | Day3 | Day4 | Day5 | Day6 | Day7 | Day8 | Day9 | Day10 | Day11 | Day12 |
| 2024 | 0-5 | YC | 17.39 | 21.50 | 21.82 | 16.50 | 19.74 | 17.57 | 12.18 | 13.73 | 17.44 | 12.91 | 14.89 | 17.45 |
| | | WD | 16.37 | 20.44 | 21.33 | 15.97 | 16.38 | 15.60 | 11.94 | 13.68 | 16.71 | 12.70 | 14.48 | 17.03 |
| | | CK | 18.02 | 21.87 | 22.38 | 16.72 | 19.99 | 19.86 | 14.15 | 17.19 | 17.97 | 15.02 | 17.83 | 19.90 |
| | 5-10 | YC | 16.26 | 20.41 | 21.25 | 16.08 | 18.55 | 15.78 | 12.14 | 13.67 | 16.67 | 12.93 | 14.41 | 17.07 |
| | | WD | 14.61 | 17.71 | 19.33 | 15.66 | 16.76 | 15.93 | 13.13 | 13.21 | 15.31 | 13.20 | 14.24 | 16.22 |
| | | CK | 16.52 | 20.50 | 21.34 | 17.07 | 18.44 | 19.64 | 14.62 | 15.82 | 17.03 | 17.22 | 17.22 | 17.34 |
| | 10-15 | YC | 15.39 | 17.04 | 19.24 | 17.12 | 17.39 | 17.10 | 15.23 | 15.26 | 17.10 | 15.05 | 15.66 | 17.62 |
| | | WD | 13.78 | 15.36 | 17.28 | 15.91 | 15.97 | 15.84 | 14.49 | 14.12 | 15.24 | 14.36 | 14.46 | 15.50 |
| | | CK | 15.77 | 17.81 | 20.13 | 17.17 | 17.23 | 17.16 | 15.03 | 15.54 | 17.22 | 14.93 | 15.91 | 17.83 |
| | 15-20 | YC | 14.90 | 16.21 | 18.05 | 17.94 | 17.82 | 17.24 | 14.67 | 15.21 | 16.34 | 15.38 | 15.38 | 16.68 |
| | | WD | 13.58 | 14.89 | 16.71 | 15.93 | 15.79 | 15.86 | 15.02 | 14.50 | 15.40 | 14.73 | 14.63 | 15.49 |
| | | CK | 14.66 | 15.90 | 17.98 | 18.97 | 17.72 | 17.06 | 16.98 | 14.73 | 15.66 | 16.67 | 14.91 | 15.90 |

Table S6 Effects of relay cropping green manure on soil daily average relative humidity.

| Year | Soil depth（cm） | Treatment | Humidity（%） | | | | | | | | | | | |
|---|---|---|---|---|---|---|---|---|---|---|---|---|---|---|
| | | | Day1 | Day2 | Day3 | Day4 | Day5 | Day6 | Day7 | Day8 | Day9 | Day10 | Day11 | Day12 |
| 2023 | 0-5 | YC | 41.29 | 43.72 | 45.69 | 43.58 | 45.40 | 48.18 | 49.10 | 47.20 | 43.59 | 46.36 | 49.68 | 45.68 |
| | | WD | 68.45 | 69.50 | 68.36 | 78.56 | 75.00 | 76.90 | 76.17 | 75.02 | 76.86 | 79.37 | 76.77 | 74.98 |
| | | CK | 60.78 | 64.15 | 62.15 | 65.81 | 67.02 | 69.67 | 62.34 | 64.10 | 62.81 | 65.14 | 66.07 | 67.32 |
| | 5-10 | YC | 41.14 | 44.35 | 40.28 | 44.35 | 46.49 | 42.42 | 44.70 | 45.41 | 41.52 | 41.23 | 46.40 | 41.04 |
| | | WD | 72.57 | 73.57 | 67.86 | 72.90 | 72.00 | 73.93 | 73.72 | 69.65 | 69.28 | 76.89 | 71.45 | 72.76 |
| | | CK | 70.59 | 73.10 | 66.20 | 70.17 | 79.57 | 70.04 | 75.30 | 70.10 | 68.33 | 77.88 | 73.86 | 64.29 |
| | 10-15 | YC | 51.91 | 54.91 | 48.71 | 54.79 | 55.13 | 50.98 | 54.37 | 55.78 | 50.88 | 50.46 | 56.62 | 49.14 |
| | | WD | 73.61 | 76.00 | 68.05 | 74.90 | 77.00 | 73.82 | 72.87 | 79.54 | 79.83 | 75.00 | 79.77 | 71.56 |
| | | CK | 63.96 | 67.86 | 63.32 | 68.94 | 70.00 | 66.49 | 68.43 | 66.24 | 62.79 | 69.85 | 69.35 | 61.04 |
| | 15-20 | YC | 63.28 | 68.97 | 63.13 | 68.68 | 68.17 | 68.64 | 68.17 | 65.82 | 64.26 | 68.33 | 67.69 | 64.91 |
| | | WD | 66.58 | 68.20 | 63.27 | 69.61 | 70.00 | 69.36 | 66.12 | 66.04 | 66.84 | 68.70 | 68.70 | 68.45 |
| | | CK | 61.42 | 64.39 | 55.09 | 65.47 | 69.15 | 64.23 | 62.93 | 62.61 | 60.27 | 61.16 | 63.04 | 61.83 |

| Year | Soil depth（cm） | Treatment | Humidity（%） | | | | | | | | | | | |
|---|---|---|---|---|---|---|---|---|---|---|---|---|---|---|
| | | | Day1 | Day2 | Day3 | Day4 | Day5 | Day6 | Day7 | Day8 | Day9 | Day10 | Day11 | Day12 |
| 2024 | 0-5 | YC | 41.29 | 41.14 | 51.91 | 83.28 | 88.45 | 92.57 | 98.61 | 71.58 | 58.78 | 80.59 | 73.96 | 71.42 |
| | | WD | 43.72 | 44.35 | 54.91 | 88.97 | 99.50 | 93.57 | 100.00 | 78.20 | 64.15 | 83.10 | 77.86 | 74.39 |
| | | CK | 45.69 | 40.28 | 48.71 | 87.13 | 80.36 | 87.86 | 88.05 | 73.27 | 62.15 | 76.20 | 73.32 | 60.09 |
| | 5-10 | YC | 43.58 | 44.35 | 54.79 | 88.68 | 98.56 | 92.90 | 99.90 | 79.61 | 65.81 | 80.17 | 78.94 | 75.47 |
| | | WD | 45.40 | 46.49 | 55.13 | 88.17 | 95.00 | 92.00 | 100.00 | 80.00 | 67.02 | 89.57 | 80.00 | 79.15 |
| | | CK | 48.18 | 42.42 | 50.98 | 88.64 | 96.90 | 98.93 | 93.82 | 79.36 | 69.67 | 76.04 | 76.49 | 74.23 |
| | 10-15 | YC | 49.10 | 44.70 | 54.37 | 88.17 | 96.17 | 93.72 | 97.87 | 76.12 | 62.34 | 85.30 | 78.43 | 72.93 |
| | | WD | 47.20 | 45.41 | 55.78 | 85.82 | 95.02 | 89.65 | 99.54 | 76.04 | 64.10 | 80.10 | 76.24 | 72.61 |
| | | CK | 43.59 | 41.52 | 50.88 | 84.26 | 96.86 | 89.28 | 99.83 | 76.84 | 62.81 | 78.33 | 72.79 | 70.27 |
| | 15-20 | YC | 46.36 | 41.23 | 50.46 | 88.33 | 99.37 | 96.89 | 100.00 | 88.70 | 65.14 | 87.88 | 79.85 | 71.16 |
| | | WD | 49.68 | 46.40 | 56.62 | 87.69 | 96.77 | 91.45 | 99.77 | 78.70 | 66.07 | 83.86 | 79.35 | 73.04 |
| | | CK | 45.68 | 41.04 | 49.14 | 84.91 | 94.98 | 92.76 | 91.56 | 78.45 | 67.32 | 64.29 | 81.04 | 71.83 |

Figure S1 PLS-DA analysis of volatile aroma substances of berris in 2023. a:Replacement test, b:biplot, c:VIP

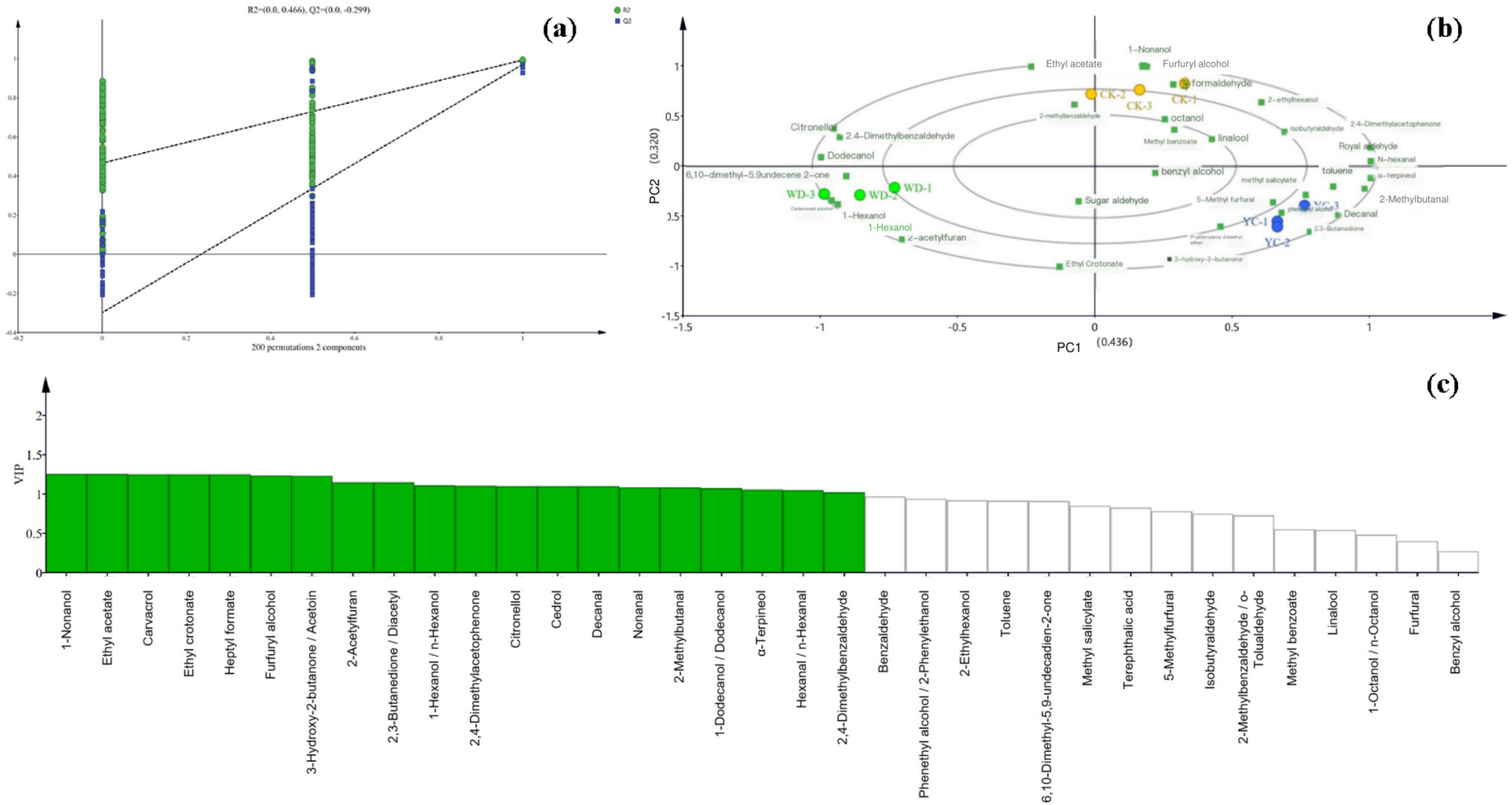

Figure S2 PLS-DA analysis of volatile aroma substances of berris in 2024. a:Replacement test, b:biplot, c:VIP

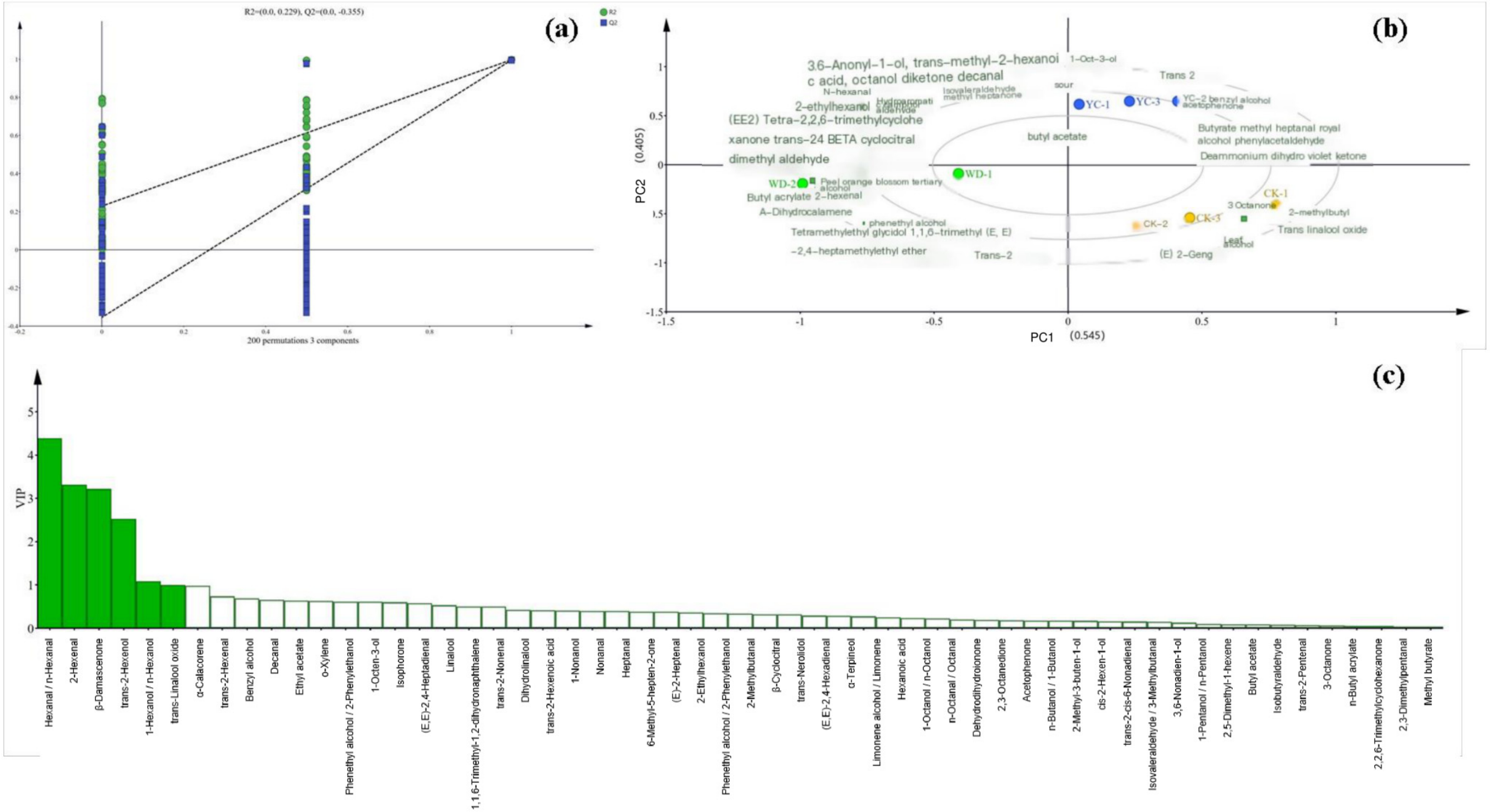

Figure S3 PLS-DA analysis of volatile aroma substances of wine in 2023. a:Replacement test, b:biplot, c:VIP

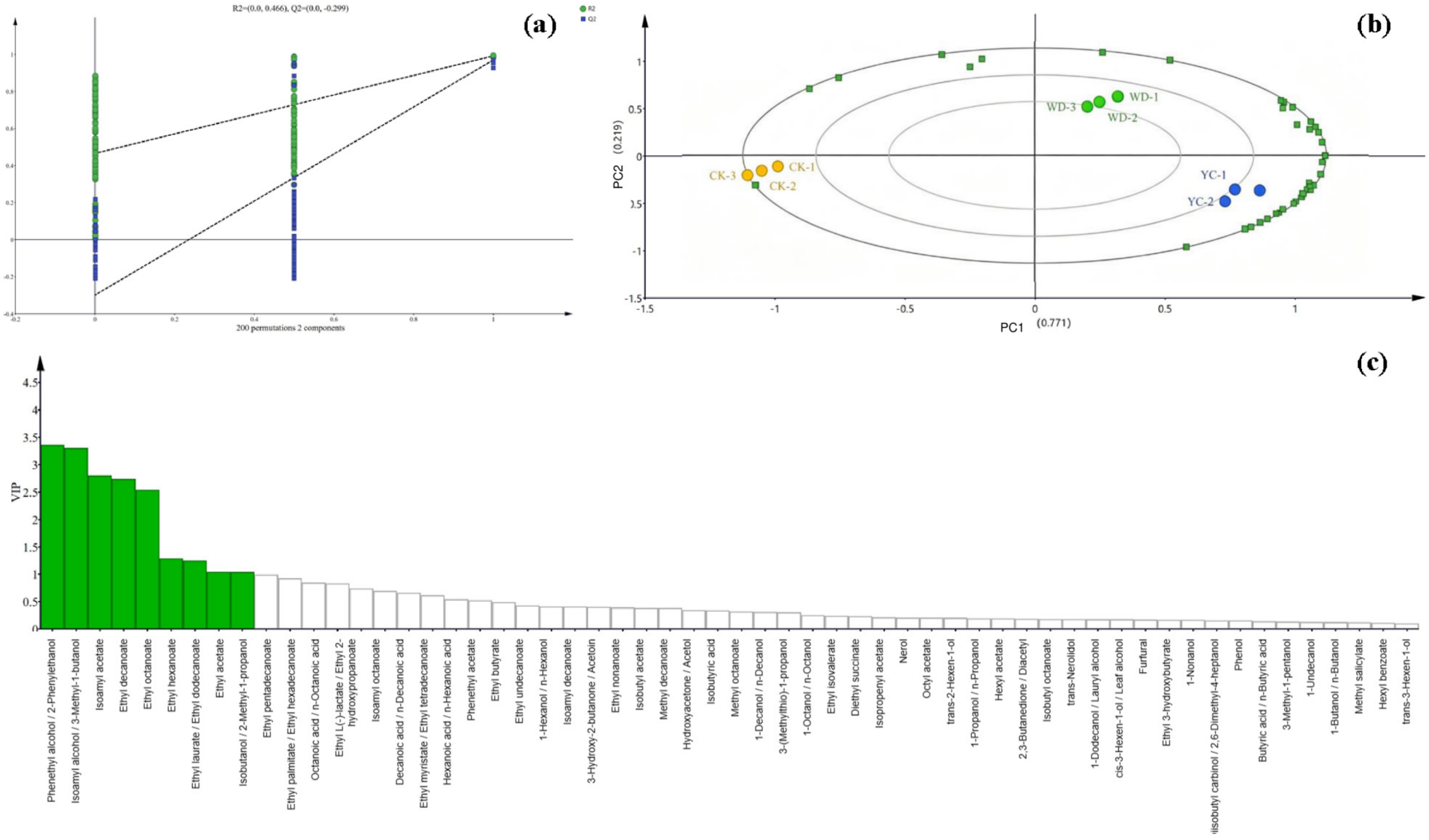

Figure S4 PLS-DA analysis of volatile aroma substances of wien in 2024. a:Replacement test, b:biplot, c:VIP

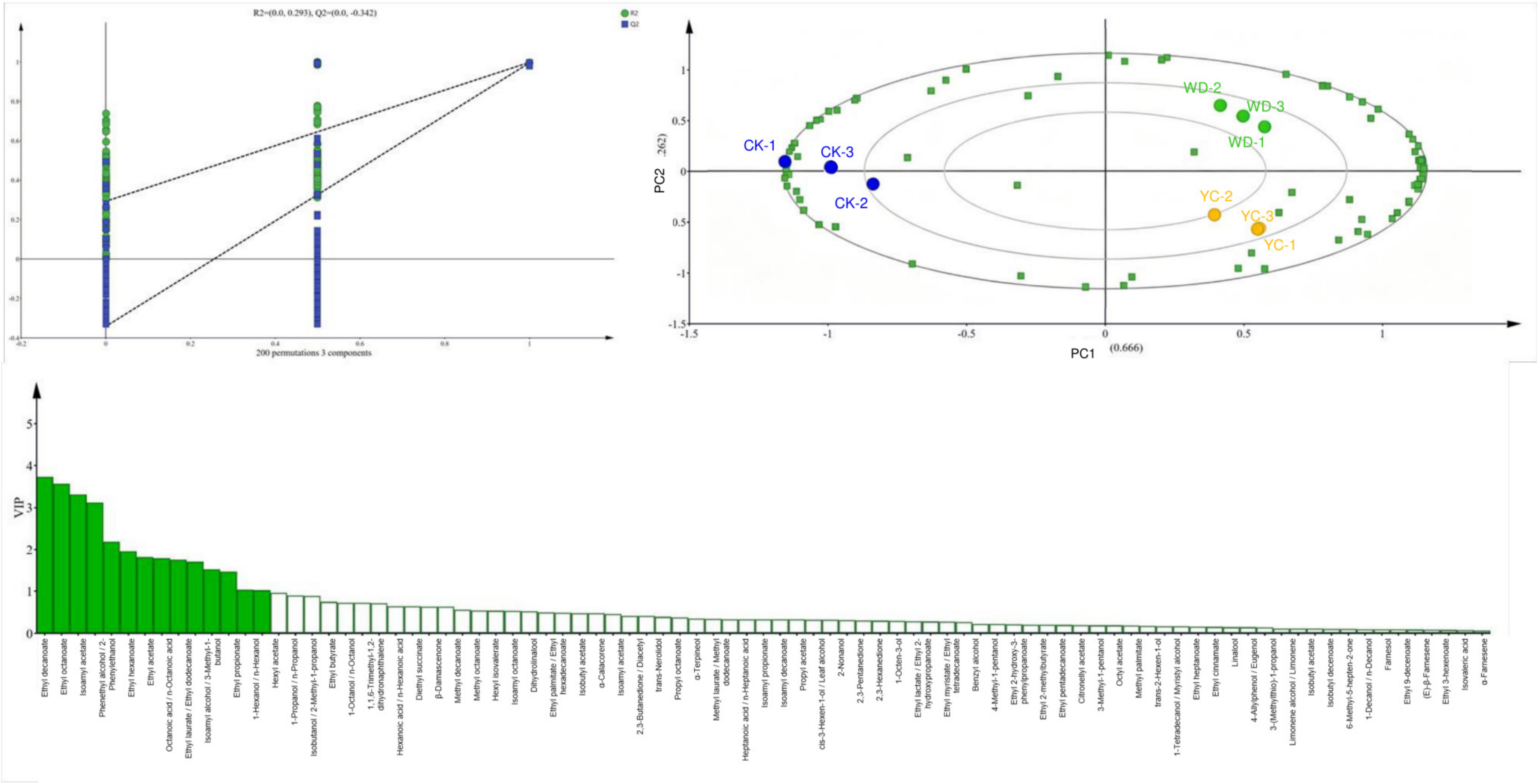

Figure S5 LEfSe analysis of soil microbial communities under different treatments.

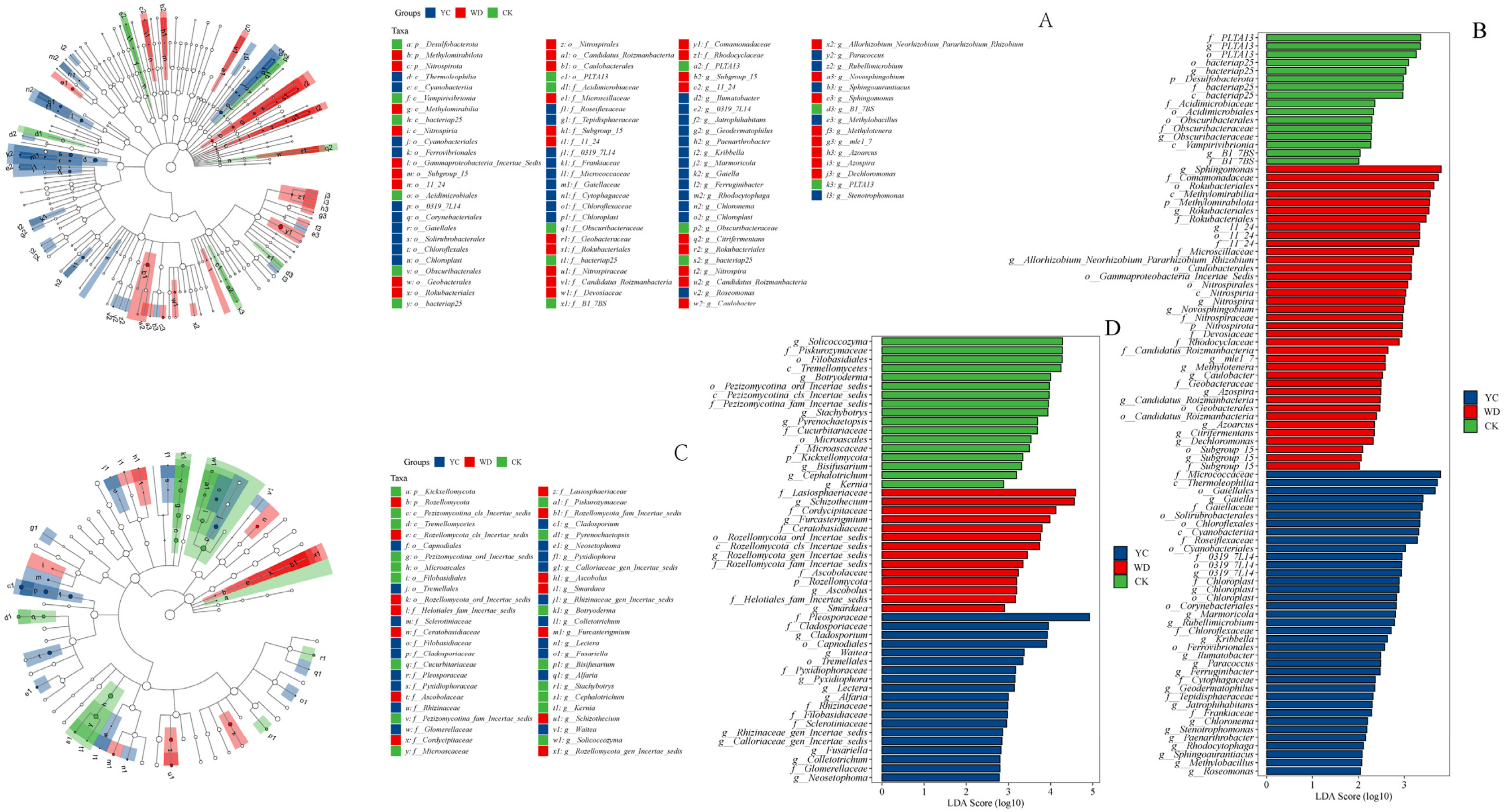

Table S7. Principal component analysis of grape berry quality under different treatments.

| Index | 2023 | | 2024 | |
|---|---|---|---|---|
| | PC1 | PC2 | PC1 | PC2 |
| $x_1$： Hundred - grain weight | 0.91 | -0.42 | 0.08 | 1.00 |
| $x_2$： Fruit shape index | -0.99 | -0.17 | 0.84 | -0.55 |
| $x_3$： pH | 0.94 | -0.35 | 0.40 | 0.92 |
| $x_4$： Soluble solid content | -0.75 | 0.66 | 0.53 | 0.85 |
| $x_5$： Reducing sugar content | -0.54 | 0.84 | 0.49 | 0.87 |
| $x_6$： Content of titrable acidity | -0.94 | 0.35 | 0.18 | -0.98 |
| $x_7$： Total phenols | 0.45 | 0.90 | 1.00 | -0.10 |
| $x_8$： Total tannins | 0.63 | 0.78 | 1.00 | 0.00 |
| $x_9$： Total anthocyanins | -0.06 | 1.00 | 0.87 | -0.49 |
| $x_{10}$： Total flavonoids | 1.00 | -0.01 | -0.94 | -0.34 |
| $x_{11}$： Total flavanols | 0.66 | 0.75 | -0.98 | 0.21 |
| $x_{12}$： Total aroma | -0.71 | -0.70 | 0.96 | -0.29 |
| Eigenvalue | 6.94 | 5.06 | 6.93 | 5.07 |
| Contribution rate (%) | 66.8 | 22.5 | 53.1 | 40.3 |
| Cumulative contribution rate (%) | 89.3 | | 93.4 | |

Table S8 Principal component analysis of wine quality under different treatments.

| Index | 2023 | | 2024 | |
|---|---|---|---|---|
| | PC1 | PC2 | PC1 | PC2 |
| $z_1$: Alcohol | 0.30 | 0.96 | -0.94 | 0.36 |
| $z_2$: TA | -0.92 | 0.38 | 0.98 | 0.20 |
| $z_3$: pH | 0.97 | -0.26 | -1.00 | -0.05 |
| $z_4$: RS | -0.96 | -0.29 | -0.86 | 0.52 |
| $z_5$: VAC | -0.64 | 0.77 | -0.76 | 0.65 |
| $z_6$: Total phenols | -0.48 | -0.88 | 0.41 | 0.91 |
| $z_7$: Total tannins | 0.98 | 0.20 | 0.95 | 0.31 |
| $z_8$: Total anthocyanins | -0.99 | 0.17 | 0.40 | 0.92 |
| $z_9$: Total flavonoids | 0.83 | 0.56 | 0.83 | -0.57 |
| $z_{10}$: Total flavanols | 0.88 | -0.48 | -0.09 | -1.00 |
| $z_{11}$: Total aroma | 1.00 | 0.05 | - | - |
| Eigenvalue | 7.82 | 3.18 | 6.89 | 4.12 |
| Contribution rate (%) | 92.3 | 3.9 | 78.7 | 18.6 |
| Cumulative contribution rate (%) | 96.2 | | 97.3 | |
